# GATA6-CRT axis promotes stress-associated autophagy, EMT, and stemness-associated traits in pancreatic cancer

**DOI:** 10.1038/s41419-026-08914-8

**Published:** 2026-06-04

**Authors:** Ming-qing Wang, Yang Liu, Bo-yuan Chan, Hong-jia Wu, Wan-xiang Zhang, Zhong-yang Wang, Qi-long Geng, Run-xin Chang, Weijie Wang, Shu-yuan Zhang, Fu-qiang Zu, Guodong Cao, Bernhard W. Renz, Jing-tong Tang, Hua-Qin Wang, Weiwei Sheng

**Affiliations:** 1 Department of General Surgery, the First Affiliated Hospital of Anhui Medical University, Hefei, China; 2 Department of Clinical Medicine, The First Clinical College, Anhui Medical University, Hefei, China; 3 Department of Gastrointestinal Surgery, the First Hospital, China Medical University, Shenyang, China; 4 Department of Immunology, School of Basic Medical Sciences, Anhui Medical University, Hefei, China; 5 Department of General Surgery, the Second Affiliated Hospital of Anhui Medical University, Hefei, China; 6 Department of General, Visceral, and Transplant Surgery, Ludwig-Maximilians-University Munich, Munich, Germany; 7 German Cancer Consortium (DKTK), German Cancer Research Centre (DKFZ), Heidelberg, Germany; 8 Department of Biochemistry & Molecular Biology, China Medical University, Shenyang, China; 9 Key Laboratory of Cell Biology, Ministry of Public Health, and Key Laboratory of Medical Cell Biology, Ministry of Education, China Medical University, Shenyang, China

**Keywords:** Cancer stem cells, Autophagy

## Abstract

Pancreatic cancer (PC) remains a major therapeutic challenge because of its profound plasticity and adaptability. Although our previous studies established that calreticulin (CRT) promotes epithelial-mesenchymal transition (EMT), its role in linking endoplasmic reticulum stress (ERS), autophagy-associated responses, and tumor cell plasticity has remained incompletely defined. The functional role of CRT was examined using in vitro and in vivo PC models, together with CRISPR/Cas9-mediated gene silencing, overexpression approaches, and pharmacological modulation of ERS and autophagy. Clinical relevance was evaluated in human PC specimens and correlated with patient survival. Thapsigargin-induced ERS activated autophagy-associated signaling through the PERK/eIF2α-ATG5/ATG12/LC3B axis and promoted EMT in a CRT-dependent manner. Under serum-free conditions, CRT was required for AMPK/mTOR/ULK1-associated autophagy activation and stemness-associated traits. Mechanistically, CRT interacted with LC3 through a conserved LC3-interacting region (LIR; WDFL), and this interaction contributed to stress-associated autophagy and malignant phenotypes. Furthermore, GATA6 was identified as a direct transcriptional activator of CRT, defining a GATA6-CRT regulatory axis. In vivo, targeting this axis through CRT silencing or autophagy inhibition by chloroquine or ATG5 knockdown suppressed tumor growth and metastasis. Clinically, high CRT expression was associated with GATA6, LC3B, and markers linked to stemness and EMT, as well as poor prognosis. Together, these findings support a model in which the GATA6-CRT axis functions as an important stress-responsive regulator associated with autophagy, phenotypic plasticity, and aggressiveness in PC, and nominate this axis as a potential therapeutic vulnerability while highlighting the need for further work to define its full mechanistic scope.

## Introduction

Pancreatic cancer (PC) remains one of the most lethal human malignancies, with an overall five-year relative survival rate of only approximately 13% [1, 2]. This dismal prognosis is largely attributable to diagnosis at advanced stages, early and extensive metastatic dissemination, and profound therapeutic resistance. A central biological feature underlying these clinical challenges is the phenotypic plasticity of pancreatic ductal adenocarcinoma (PDAC) cells, which permits dynamic interconversion among epithelial, mesenchymal, and stem-like states [3]. Defining the molecular regulators of this plasticity is therefore essential for the development of more effective therapeutic strategies.

The endoplasmic reticulum (ER) is a central hub for protein folding and cellular homeostasis. Accumulation of misfolded proteins perturbs ER function, triggering endoplasmic reticulum stress (ERS) and the adaptive unfolded protein response (UPR) to restore proteostasis [4–6]. In cancer, however, malignant cells can co-opt the UPR to survive under adverse conditions. Chronic, sublethal ERS may fuel invasion, metabolic adaptation, and therapy resistance, in part by activating pro-survival pathways such as autophagy [7–9]. This conserved lysosomal degradation process enables tumor cells to recycle cellular components and mitigate metabolic stresses within the tumor microenvironment, including nutrient deprivation and hypoxia [8, 9]. A growing body of evidence now positions the ERS-autophagy axis as a critical contributor to EMT, stemness acquisition, and chemoresistance across diverse cancers [10–13].

Autophagy has also been implicated in the maintenance of cancer stem cells (CSCs), a subpopulation characterized by self-renewal capacity and metabolic flexibility that contributes to tumor initiation, recurrence, and metastatic outgrowth [12–14]. Despite these advances, the molecular mechanisms that integrate ERS, autophagic flux, and CSC-associated phenotypes in PDAC remain incompletely defined.

Calreticulin (CRT), a multifunctional ER chaperone, is a pivotal regulator of calcium homeostasis and protein folding [15, 16]. Our prior work established that CRT, particularly in its cytoplasmic localization, drives EMT in PDAC through integrin/EGFR-ERK/MAPK signaling and calcium-dependent ERS activation [17–19]. Given the established roles of both CRT and autophagy in PDAC adaptation, we hypothesized that CRT may function as an important molecular link connecting ER proteostasis to autophagy-driven phenotypic remodeling.

Here, we describe a GATA6-CRT regulatory axis in which the transcription factor GATA6 transcriptionally upregulates CRT, and CRT in turn contributes to the linkage among ERS, autophagy-associated responses, EMT, and stemness-associated phenotypes. We show that CRT promotes EMT in the setting of ERS-associated autophagy via the PERK/eIF2α pathway and supports stemness-associated traits through AMPK/mTOR/ULK1 signaling under nutrient stress. We further provide evidence that CRT interacts with LC3 through a conserved LIR motif, although the precise cell-biological topology of this interaction remains to be clarified. Collectively, our findings identify the GATA6-CRT axis as an important regulator of stress adaptation and tumor cell plasticity in PC and suggest that this pathway may represent a therapeutic opportunity.

## Materials and methods

### Ethics statement

This study was conducted in accordance with the Declaration of Helsinki and was approved by the Academic Committee of the First Affiliated Hospital of Anhui Medical University and China Medical University. Written informed consent was obtained from all participants before sample collection. All methods involving human participants were performed in accordance with the relevant guidelines and regulations.

### Patient samples and cell culture

A total of 102 surgically resected pancreatic ductal adenocarcinoma (PDAC/PC) tissues and paired adjacent normal tissues were obtained from the Department of General Surgery, the First Affiliated Hospital of Anhui Medical University, and the First Hospital of China Medical University. Patients with endocrine carcinoma, acinar cell carcinoma, or invasive intraductal papillary mucinous carcinoma were excluded. The human PDAC cell lines Capan-2 (HTB-80), AsPC-1 (CRL-1682), and BxPC-3 (SCSP-529), the murine PDAC line Panc02 (CRL-2553), and HEK293T cells (SCSP-502) were acquired from the American Type Culture Collection (ATCC) or the National Collection of Authenticated Cell Cultures (Shanghai, China). Cell lines were authenticated by short tandem repeat profiling and tested negative for mycoplasma before and during experimental use, with routine testing performed every 4 weeks. All cell lines were maintained in RPMI-1640 or DMEM supplemented with 10% fetal bovine serum at 37 °C in a humidified atmosphere containing 5% CO_2_. This panel was selected to provide complementary biological contexts for dissecting the GATA6-CRT-autophagy axis: Capan-2 and Panc02 cells enabled analysis of ERS-induced autophagy across species and genetic backgrounds; AsPC-1 and BxPC-3 cells, which retain a relatively epithelial phenotype, were used to explore CRT-dependent autophagy and stemness-associated traits; and HEK293T cells were used for luciferase reporter assays.

### Bioinformatic analysis

Single-cell RNA sequencing (scRNA-seq) data from three PDAC samples were processed and annotated as previously described [20]. Epithelial subclusters were defined based on EPCAM expression. Differentially expressed genes (DEGs) from this cohort (sncRNA_DEGs) were intersected with DEGs from the TCGA PDAC cohort (tumor vs. normal). A prognostic risk model was constructed using a machine-learning framework based on the overlapping genes and validated in independent TCGA and E-MTAB datasets. Survival analysis was performed using Kaplan–Meier curves and receiver operating characteristic (ROC) analysis. Functional enrichment was carried out using Cluster Profiler.

### Plasmids and CRISPR/Cas9-mediated gene silencing

Lentiviral vectors encoding CRISPR/Cas9 components and sgRNAs targeting human CRT/CALR (sgCRT1 and sgCRT2) and mouse Crt/Calr (sgCRT1), together with a non-targeting control, were synthesized by GeneChem. Stable CRT-silenced Capan-2 and Panc02 cell lines were established by lentiviral transduction followed by puromycin selection. Cells were transduced with lentiviral particles at empirically optimized multiplicities of infection (MOIs). Initial titration was performed at MOIs of 5, 10, and 20, and the final MOI was selected according to transduction efficiency and cell viability. For interaction mapping, GST-tagged wild-type CRT (GST-CRT), LIR-motif-deleted CRT (CRT-delta LIR), and His-tagged LC3 (His-LC3) constructs were generated. Target sequences for sgCRT1, sgCRT2, GATA6, ZBTB26, ATG5, and scramble controls are listed in Supplementary Table 1. siRNAs and plasmids were transfected using Lipofectamine 3000 (Invitrogen, Carlsbad, CA, USA) according to the manufacturer’s instructions.

### qRT-PCR

Using protocols from our previous studies, mRNA from PC tissues and cell lines was analyzed using the LightCycler system and Applied Biosystems 7500 Fast Real-Time PCR System. Primer sequences are listed in Supplementary Table 2. PCR product quality was monitored by post-PCR melt-curve analysis. Relative expression levels were calculated using the 2^−ΔΔCt^ method.

### Western blotting, co-immunoprecipitation and GST pull-down assay

Cells were lysed, and proteins were separated by SDS-PAGE, transferred to PVDF membranes, and probed with primary antibodies overnight at 4 °C, followed by HRP-conjugated secondary antibodies. Signals were detected using enhanced chemiluminescence. For co-immunoprecipitation (Co-IP), cell lysates were incubated with antibodies against CRT, LC3B, or control IgG pre-bound to magnetic beads. Precipitates were washed and analyzed by western blotting. The following primary antibodies were used: CRT (Abcam, ab22683), phospho-PERK (Thr980; Cell Signaling Technology [CST], #3179), phospho-eIF2α (Ser51; CST, #3597), phospho-ULK1 (Ser317; CST, #37762), ATG5 (Proteintech, 10181-2-AP), ATG12 (Proteintech, 11122-1-AP), phospho-AMPK (Thr172; CST, #2535), phospho-mTOR (Ser2448; CST, #5536), LC3B (Abmart, #T55992), ZEB1 (Proteintech, 21544-1-AP), E-cadherin (Abcam, ab231303), N-cadherin (Proteintech, 13769-1-AP), Slug (CST, #80121), phosphorylated GSK-3β (Ser9; CST, #D85E12), SOX2 (Proteintech, 66411-1-Ig), CD133 (Proteintech, 18470-1-AP), CD44 (Proteintech, 15675-1-AP), GATA6 (CST, #5851), and GAPDH (Proteintech, 60004-1-Ig). GST pull-down assays were performed using GSH-Sepharose beads, and precipitated proteins were analyzed by western blotting using anti-GST (Proteintech, 10000-0-AP) and anti-His (Proteintech, 66005-1-Ig) antibodies.

### Immunohistochemistry and immunofluorescence

For immunohistochemistry (IHC), 4-μm tissue sections underwent antigen retrieval, blocking, and incubation with primary antibodies against CRT (Abcam, ab22683), phospho-eIF2α (Ser51; CST, #3597), ATG5 (Proteintech, 10181-2-AP), LC3B (Proteintech, 14600-1-AP), phospho-AMPK (Thr172; CST, #2535), phospho-mTOR (Ser2448; CST, #2976), phospho-ULK1 (Ser317; Affinity, #AF2301), and GATA6 (CST, #5851). Sections were then developed using a streptavidin-HRP system and DAB chromogen. Staining was evaluated independently by three pathologists. For immunofluorescence (IF), cells were fixed, permeabilized, and incubated with primary and fluorescent secondary antibodies. Nuclei were counterstained with Hoechst 33258.

### Autophagic flux and transmission electron microscopy

Autophagic flux was detected with mRFP-GFP-LC3 (Hanbio, China). PC cells from the sgCRT1 and control groups were first transfected with the mRFP-GFP-LC3 adenoviral vector for 24 h and then cultured with TG (200 nM; Sigma, St. Louis, MO, USA) for 12 h or 4-phenylbutyric acid (4-PBA; 5 mM) for 12 h. mRFP and GFP signals in the tandem reporter were used to monitor LC3 puncta by confocal microscopy (Leica TCS SP5 II, Leica, Heidelberg, Germany). Increases in red and yellow puncta were interpreted as changes in autophagic structures and flux. Experiments were repeated at least three times. Transmission electron microscopy (TEM) was performed on PC cells in vitro and tumor tissues in vivo. Briefly, cells were digested and suspended at 5 × 106 cells/mL. Tumor tissues were cut into 1 × 1 × 3 mm^3^ strips. Cells and tissues were fixed at 4 °C for 6–12 h with 1.5% glutaraldehyde. Ultrathin sections (100 nm) were then prepared, stained with uranyl acetate and lead citrate, and examined using TEM (H-7650; Hitachi, Tokyo, Japan).

### Functional assays

Cell invasion and migration were assessed using Matrigel-coated or uncoated Transwell chambers, respectively. Spheroid formation assays were performed to assess stemness-associated properties by culturing cells in serum-free DMEM/F12 medium supplemented with B-27, N-2, epidermal growth factor, and fibroblast growth factor in low-attachment plates. Chemosensitivity to gemcitabine was determined using the MTT assay.

### Chromatin immunoprecipitation and luciferase reporter assay

Chromatin immunoprecipitation (ChIP) was performed using a commercial kit (Cell Signaling Technology). Crosslinked chromatin was sonicated and immunoprecipitated with an anti-GATA6 antibody. Precipitated DNA was analyzed by PCR. For luciferase reporter assays, HEK293T cells were co-transfected with a GATA6 expression vector and CRT promoter-luciferase constructs (wild-type or mutant). Luciferase activity was measured 48 h after transfection.

### In vivo studies

All animal experiments were approved by the Animal Care and Use Committee of Anhui Medical University and performed in accordance with institutional guidelines for the care and use of laboratory animals.

### Liver metastasis model

A total of 20 female C57BL/6 mice (8 weeks old) were used. Mouse Panc02 cells (1 × 10^6^ cells suspended in 100 μL PBS) were injected into the spleen to establish an experimental liver metastasis model. Mice were randomly assigned to the indicated treatment groups, including vehicle control, TG, TG plus CRT silencing (sgCRT), and TG plus chloroquine (CQ). Investigators responsible for outcome assessment, including quantification of metastatic nodules, histopathological evaluation, and biochemical analyses, were blinded to group allocation.

### Subcutaneous tumor model

A total of 20 female C57BL/6 mice (8 weeks old) were used. Panc02 cells (1 × 10^6^ cells in 50 μL PBS mixed with 50 μL Matrigel) were subcutaneously implanted into the axillary region. Mice were randomly assigned to the scramble, sgCRT, sgCRT + CRT-delta LIR, and sgCRT + CRT overexpression (CRT-OE) groups under TG treatment.

### In vivo tumor-initiating and serial transplantation assay

To assess tumor-initiating capacity, AsPC-1-derived sphere cells from the scramble, shATG5, CRT-OE, and CRT-OE + shATG5 groups were subcutaneously implanted into the axillary region of NOD-SCID mice. At the experimental endpoint, primary tumors derived from sphere cells were aseptically excised and mechanically dissociated into small fragments. Tumor tissues were enzymatically digested with collagenase, hyaluronidase, and DNase I or processed into single-cell suspensions according to standard protocols. After filtration through a 40-μm cell strainer and viability assessment, viable tumor cells were counted and prepared for secondary transplantation using the same group design (*n* = 5 per group). At the second endpoint, mice were euthanized, and subcutaneous tumors were harvested for tumor burden measurement, histological analysis, and subsequent transplantation assays.

### Orthotopic pancreatic tumor model

A total of 15 female NOD-SCID mice (8 weeks old) were used. AsPC-1-derived sphere cells were surgically implanted into the pancreas to establish orthotopic tumors. Mice were randomly assigned using a computer-generated randomization method to the scramble, CRT overexpression (CRT-OE/CRT), and CRT + CQ treatment groups. Tumor growth and metastatic dissemination were monitored throughout the study. At the experimental endpoint, mice were euthanized, and primary tumors and metastatic tissues were collected for histopathological and biochemical analyses.

### Statistical analysis

Data are presented as the mean ± standard deviation (SD) unless otherwise indicated. Statistical comparisons were performed using SPSS 21.0. Two-group comparisons were analyzed using unpaired two-tailed Student’s *t*-tests when data were normally distributed; non-parametric data were analyzed using the Mann–Whitney *U* test. Multiple-group comparisons were analyzed using one-way ANOVA followed by appropriate post hoc tests. Correlations were assessed using Spearman correlation tests. Survival data were analyzed using the Kaplan–Meier method and log-rank test, and Cox proportional hazards regression was used for prognostic analyses. The exact *n* values, definitions of biological replicates, error bars, and statistical symbols are provided in the corresponding figure legends. A two-sided *P* value < 0.05 was considered statistically significant.

## Results

### A multi-omics prognostic model identifies calreticulin as a key mediator in PC

**Fig. 1:**
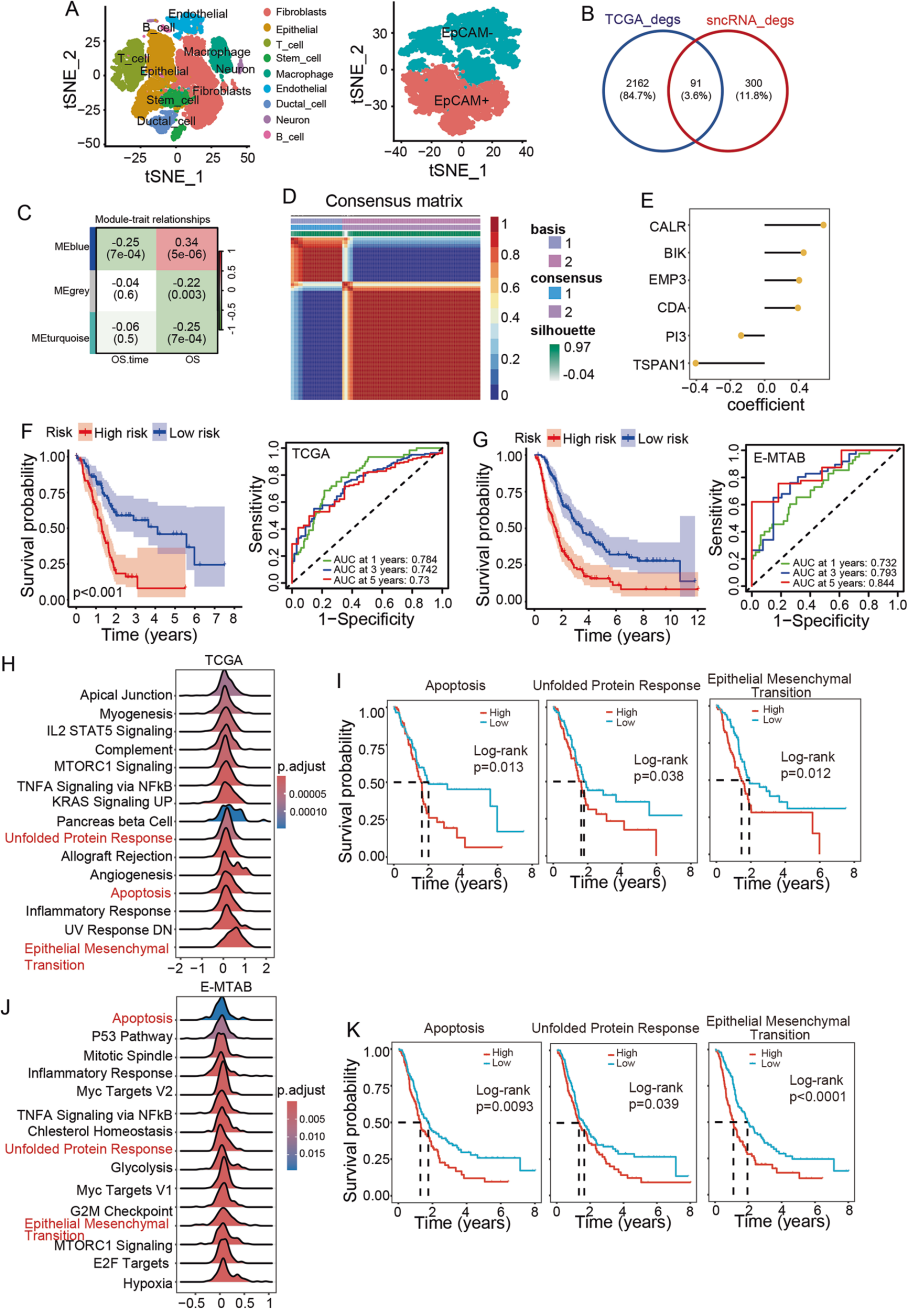
Identification of a prognostic risk model in pancreatic cancer. **A** t-SNE distribution from scRNA-seq analysis showing nine distinct clusters and cell-type annotation of epithelial subclusters. **B** Overlapping genes identified between TCGA and the single-nucleus RNA-seq cohort. **C** Correlation heatmap showing associations of three gene modules with overall survival time and status. **D** Optimal stratification of risk scores determined by the NFM algorithm. **E** Selection of prognostic genes for the risk model using machine-learning approaches. **F** Survival analysis comparing high- and low-risk groups and ROC curves assessing model performance in the TCGA cohort. **G** Validation of survival differences and corresponding ROC curves in the E-MTAB cohort. **H**, **I** Functional enrichment analysis of autophagy-related genes and survival analysis stratified by apoptosis, unfolded protein response, and EMT in TCGA. **J**, **K** Functional enrichment analysis of autophagy-related genes and survival analysis based on apoptosis, unfolded protein response, and EMT in the E-MTAB cohort.

To systematically identify key drivers of PC progression, we integrated scRNA-seq data with bulk transcriptomic profiles from The Cancer Genome Atlas (TCGA). Re-clustering of epithelial cells revealed distinct subpopulations, with EPCAM+ tumor cells exhibiting enhanced invasive potential (Fig. 1A). We identified DEGs from this scRNA-seq cohort (sncRNA_DEGs) and from PC versus normal tissues in TCGA (TCGA_DEGs), yielding 91 overlapping genes (Fig. 1B). Weighted gene co-expression network analysis (WGCNA) identified a survival-associated module (blue module, ME = 0.34, *P* = 5 × 10^−6^; Fig. 1C), from which a prognostic risk model was derived (Fig. 1D).

Using a machine-learning framework, we established a six-gene prognostic signature that included calreticulin (CRT/CALR) and validated it in independent TCGA and E-MTAB cohorts (Fig. 1E). This model effectively stratified patients into high- and low-risk groups with significantly distinct overall survival (Fig. 1F, G) and demonstrated strong predictive accuracy across 1-, 3-, and 5-year intervals (AUCs: TCGA, 0.73-0.78; E-MTAB, 0.73-0.84; Fig. 1F, G). Pathway enrichment analysis further associated the high-risk phenotype with apoptosis, the UPR, and EMT (Fig. 1H–K). Together with our prior findings on CRT in EMT and ERS [17–19], these data nominated CRT as a candidate mediator linking ERS-associated autophagy with tumor cell plasticity in PC.

### CRT regulates ER stress-associated autophagic responses in PC

**Fig. 2:**
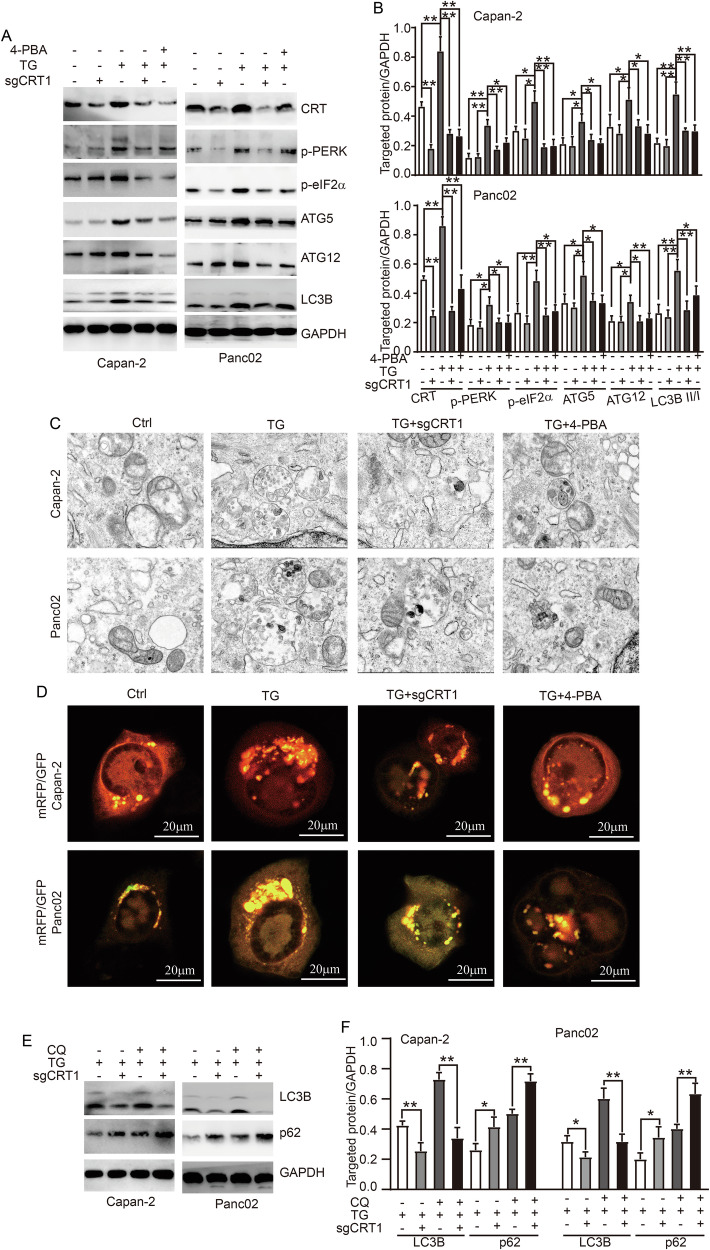
CRT silencing inhibits TG-induced ERS and autophagy signaling in PC cells. **A**, **B** Western blotting and quantification of key UPR (PERK/eIF2α) and autophagy (ATG5/ATG12/LC3B) pathway markers in CRT-silenced Capan-2 and Panc02 cells treated with or without TG and 4-PBA. CRT silencing inhibited TG-induced autophagosome formation and autophagic flux in PC cells, as assessed by TEM (**C**) and mRFP-GFP-LC3 assays (**D**). **E**, **F** Western blotting and quantification of LC3B and p62 expression in CRT-silenced Capan-2 and Panc02 cells with or without TG and CQ treatment. TG: thapsigargin, 200 nM for 12 h; CQ: chloroquine, 50 nM for 12 h; 4-PBA: 4-phenylbutyric acid, 5 mM for 12 h. Error bars represent SD. *P < 0.05; **P < 0.01 compared with control.

We first investigated the role of CRT in ERS-associated autophagy. In both Capan-2 and Panc02 PC cells, the ERS inducer TG robustly upregulated CRT, activated the PERK/eIF2α UPR axis (p-PERK and p-eIF2α), and induced autophagy markers (ATG5, ATG12, and LC3B-II). These effects were mitigated by either CRT silencing or the ERS inhibitor 4-PBA (Fig. 2A, B). Direct visualization by TEM confirmed that TG-induced autophagosome formation was markedly suppressed by CRT silencing or 4-PBA (Fig. 2C). Consistently, mRFP-GFP-LC3 reporter assays revealed that TG-enhanced autophagic responses were abrogated by CRT silencing (Fig. 2D). To further interrogate autophagy-associated flux, we used CQ, which blocks autophagosome degradation. CRT silencing combined with TG and CQ led to reduced LC3B accumulation and increased p62 accumulation compared with TG + CQ alone (Fig. 2E, F), indicating that CRT contributes to stress-induced autophagic responses under ER stress.

Because TG is a potent pharmacological stimulus that does not fully recapitulate PDAC microenvironmental stress, we further tested a more physiologically relevant ER stress condition induced by glucose insufficiency (Supplementary Fig. 1). Under low-glucose medium (LM; 1 mM), ER stress and autophagy were induced, as indicated by activation of p-PERK, p-eIF2α, GRP78, and LC3B-II, together with reduced p62; these effects were inhibited by 4-PBA (Supplementary Fig. 1a). Similarly, CRT silencing inhibited LM-induced autophagy and EMT-associated phenotypes in vitro (Supplementary Fig. 1b, c), suggesting that the role of CRT in ERS-induced autophagy is also observed under microenvironmentally relevant stress conditions.

Together, these data support CRT as an important regulator of ER stress-associated autophagic responses in PC.

### CRT couples ER stress to autophagy-associated EMT and chemoresistance

**Fig. 3:**
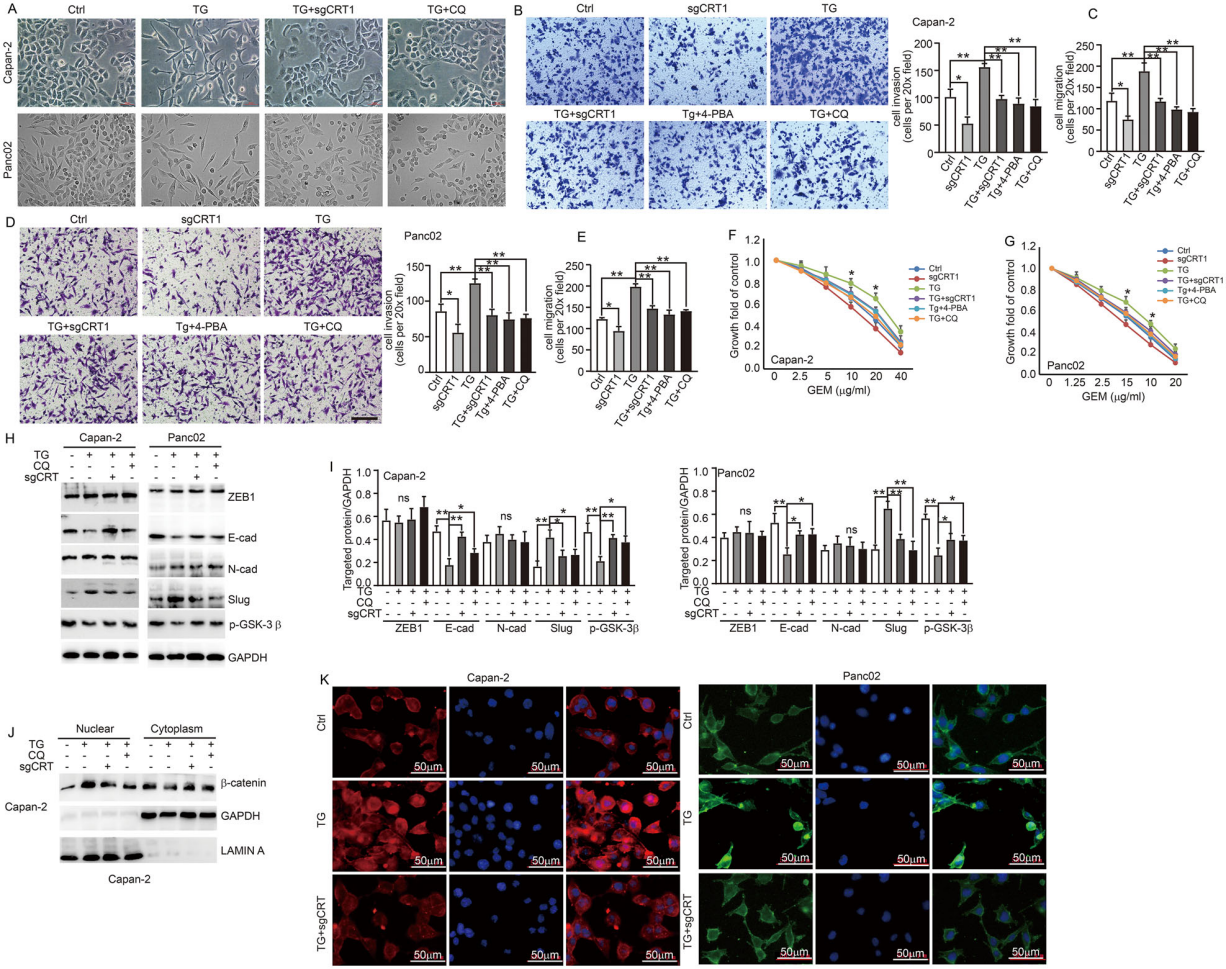
CRT silencing inhibits ERS-induced EMT by modulating autophagy in vitro. **A** Cellular morphology in Capan-2 and Panc02 cells under Ctrl, TG, TG plus sgCRT, and TG plus CQ conditions. Cell invasion (**B**) and migration (**C**) of Capan-2 cells in Ctrl, sgCRT1, TG, TG plus sgCRT1, TG plus 4-PBA, and TG plus CQ groups. **D**, **E** Corresponding invasion and migration results in Panc02 cells. Cell viability assessed by MTT in Capan-2 (**F**) and Panc02 (**G**) cells across the indicated treatment groups. **H**, **I** Western blotting and quantification of EMT markers and related signaling molecules in Ctrl, TG, TG plus sgCRT1, and TG plus CQ groups. **J** Cytoplasmic and nuclear β-catenin expression in Capan-2 cells under the same treatments, analyzed by western blotting. **K** IF staining of β-catenin in Ctrl, TG, and TG plus sgCRT1 groups in Capan-2 and Panc02 cells. Error bars represent SD. *P < 0.05; **P < 0.01 compared with control.

ERS and autophagy are tightly interconnected cellular mechanisms that work together to maintain cellular homeostasis and play a significant role in carcinogenesis [21, 22]. Here, TG treatment induced a spindle-shaped, EMT-like morphology in approximately 85% of PC cells, whereas CRT silencing or autophagy inhibition with CQ reduced this proportion to approximately 25% (Fig. 3A). Functional assays showed that TG markedly enhanced cell invasion and migration, and these effects were abolished by CRT silencing, 4-PBA, or CQ treatment (Fig. 3B–E). TG also conferred gemcitabine resistance, which was similarly reversed by targeting the CRT/autophagy axis (Fig. 3F, G).

Mechanistically, TG-triggered EMT was characterized by E-cadherin (E-cad) loss and Slug upregulation, without marked changes in ZEB1 or N-cadherin (Fig. 3H, I). CRT silencing or autophagy inhibition restored E-cadherin, suppressed Slug, and reduced GSK-3β phosphorylation. These changes were accompanied by blockade of β-catenin nuclear translocation (Fig. 3J, K), positioning the GSK-3β/β-catenin pathway downstream of CRT-mediated autophagy-associated EMT.

### CRT promotes stemness-associated traits via autophagy activation

**Fig. 4:**
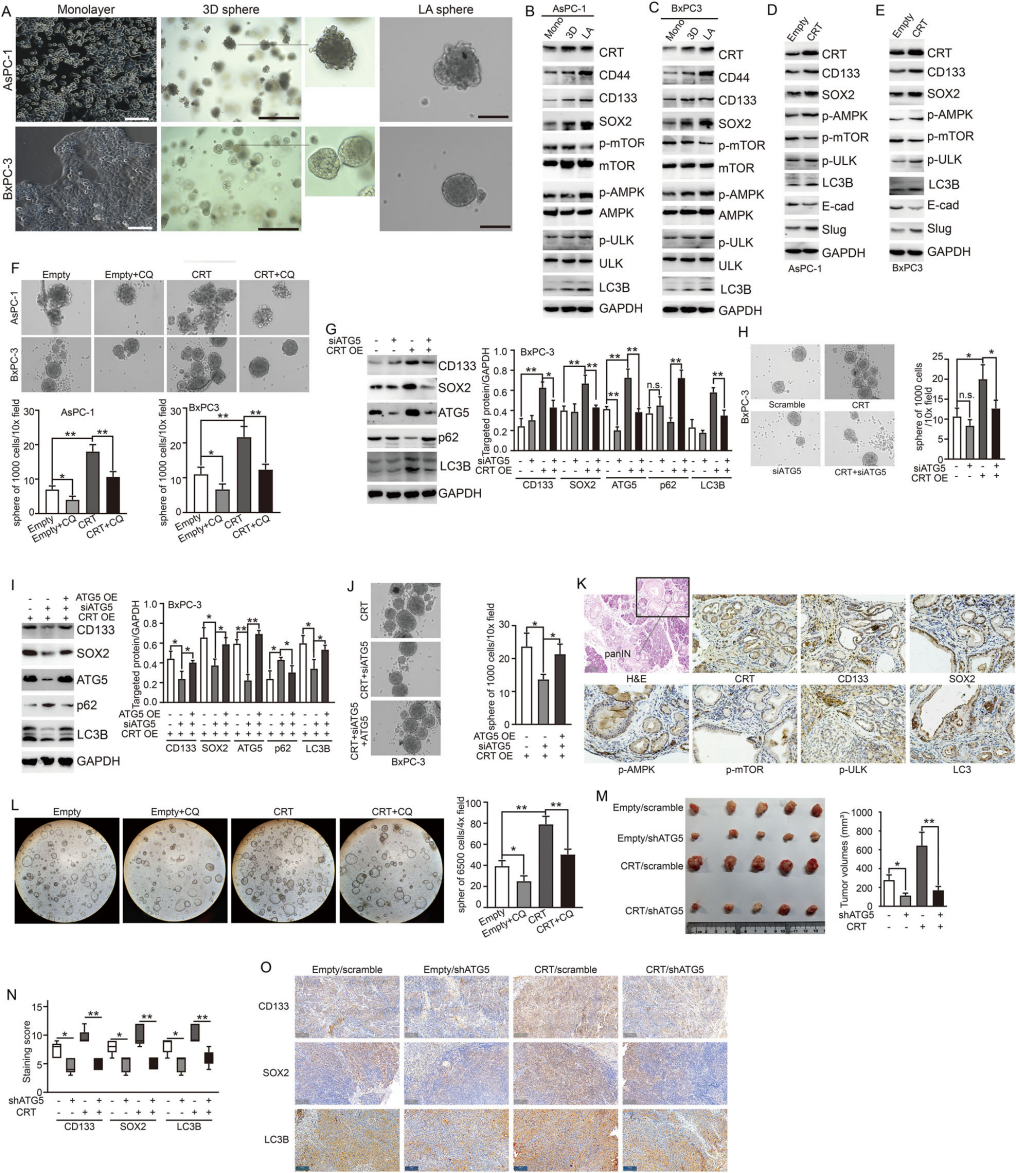
CRT promotes stemness-associated properties via autophagy activation in vitro and in vivo. **A** Schematic representation of monolayer culture and subsequent 3D and low-attachment sphere formation for stemness enrichment. Expression of CRT, stemness markers, and AMPK/mTOR/LC3B autophagy pathway components in AsPC-1 (**B**) and BxPC-3 (**C**) cells. **D**, **E** Western blot analysis of indicated proteins in CRT-OE versus Ctrl groups. **F** Sphere numbers in Ctrl, CQ, CRT-OE, and CRT-OE + CQ groups in vitro. **G** Western blotting and quantification of indicated proteins in Ctrl, siATG5, CRT-OE, and CRT-OE plus siATG5 groups in BxPC-3 cells. **H** Sphere numbers in the groups described in (**G**). **I** Western blotting and quantification of indicated proteins in CRT-OE, CRT-OE plus siATG5, and CRT-OE plus siATG5 combined with ATG5-OE groups in BxPC-3 cells. **J** Sphere numbers in the groups described in (**I**). **K** Expression of CRT, stemness markers, and autophagy-related proteins in PanIN lesions of KC mice. **L** Quantification of spheroid numbers isolated from PanIN tissues of KC, KC + CQ, KC + CRT-OE, and KC + CRT-OE + CQ groups. **M** Orthotopic transplantation of sphere-derived cells and serial transplantation assay in Ctrl, shATG5, CRT-OE, and shATG5 + CRT-OE groups. **N**, **O** Quantification and representative IHC images of CD133, SOX2, and LC3B expression described in (**M**). Error bars represent SD. *P < 0.05; **P < 0.01 compared with control.

The tumor microenvironment often imposes nutrient stress, a key trigger for autophagy [23]. Under serum-free, low-adhesion conditions that enrich for stem-like tumor cells, we observed concurrent upregulation of CRT and core stemness markers (CD44, CD133, and SOX2), together with activation of the AMPK/mTOR/ULK1 autophagy pathway (Fig. 4A–C and Supplementary Fig. 2). CRT overexpression was sufficient to enhance autophagy, increase sphere formation, and upregulate stemness-associated and EMT-associated markers (CD133, SOX2, and Slug), whereas CQ treatment negated these pro-tumorigenic effects (Fig. 4D–F and Supplementary Fig. 2). Rescue experiments in ATG5-knockdown PDAC cells further supported the autophagy dependence of these phenotypes. Similar to CQ, ATG5 knockdown significantly decreased CRT-induced stemness-associated phenotypes, including stemness marker expression and sphere numbers, and these effects were rescued by exogenous ATG5 expression (Fig. 4G–J).

In vivo, CRT and stemness- and autophagy-related markers were highly expressed in pancreatic intraepithelial neoplasia (PanIN) lesions of KC mice (Fig. 4K). CRT overexpression accelerated PanIN spheroid formation, an effect blunted by CQ (Fig. 4l). Furthermore, CRT overexpression promoted xenograft growth and expression of stemness-related markers in vivo, whereas ATG5 knockdown suppressed these effects (Fig. 4M–O). Collectively, these data indicate that CRT-driven autophagy supports stemness-associated and tumor-initiating phenotypes in vivo.

### CRT interacts with LC3 via an LIR motif and modulates autophagy-associated phenotypes

**Fig. 5:**
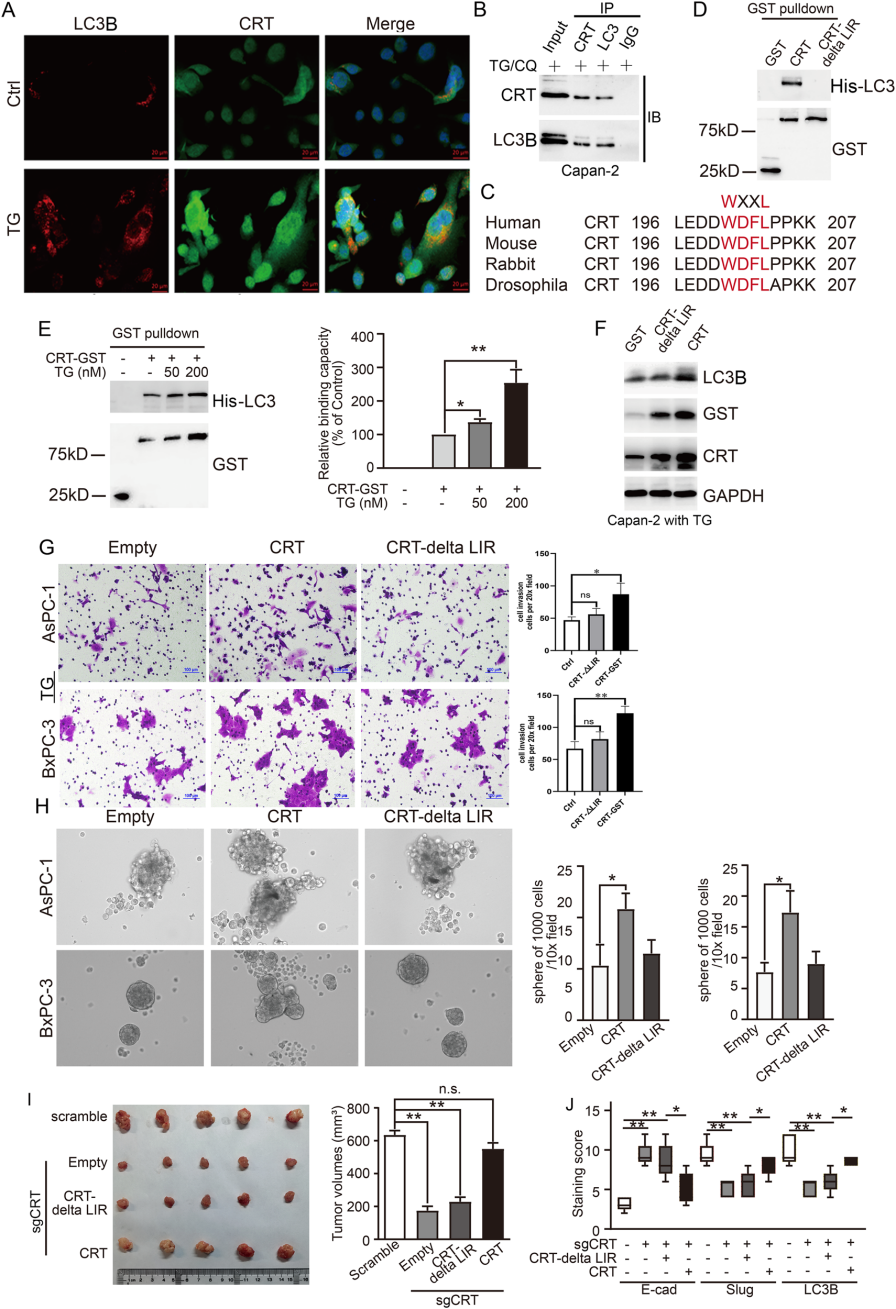
CRT interacts with LC3 via its LIR motif and contributes to ERS-associated autophagy-related phenotypes. **A** Co-localization of CRT (FITC, green) and LC3B (TRITC, red) in Capan-2 cells under TG plus CQ treatment. **B** Co-IP of CRT and LC3B in Capan-2 cells under TG plus CQ treatment. **C** Evolutionary conservation analysis of the predicted CRT LIR motif. **D** GST pull-down assay in HEK293T cells co-transfected with the indicated constructs. GST- and His-tagged proteins were pulled down using GSH-agarose beads, and co-precipitated proteins were detected by western blotting. **E** Enhanced CRT-LC3 interaction under ERS. HEK293T cells were co-transfected with the indicated constructs and treated with increasing TG concentrations (50–200 nM for 12 h), followed by GST pull-down and western blotting. **F** LC3B accumulation was not induced by CRT-delta LIR. Capan-2 cells were transfected with GST-CRT and CRT-delta LIR constructs and treated with DMSO or TG (200 nM for 12 h), followed by western blotting. Effects of CRT overexpression and LIR truncation (CRT-delta LIR) on cell invasion (**G**) and sphere formation (**H**) in vitro. **I** Subcutaneous tumor size in control, sgCRT, sgCRT + CRT-delta LIR, and sgCRT + CRT-OE groups under TG treatment. **J** Quantification of IHC staining for E-cadherin, Slug, and LC3B in the tumors described in (**I**). Error bars represent SD. *P < 0.05; **P < 0.01 compared with control.

We next investigated the molecular mechanism by which CRT contributes to autophagy. Immunofluorescence and Co-IP assays supported a stress-enhanced physical interaction between CRT and LC3B (Fig. 5A, B). The LC3-interacting region (LIR) motif is crucial for selective autophagy, ensuring the targeting of autophagy receptors to LC3/ATG8-family proteins anchored in the phagophore membrane [24]. The LIR motif contains the consensus sequence W/Y/FXXL/I/V (where X represents any residue) and is present in most LC3-interacting proteins [25]. Bioinformatic analysis identified a conserved LIR motif (WDFL, amino acids 200–204) within CRT (Fig. 5C). GST pull-down assays confirmed that wild-type GST-CRT, but not the LIR-deletion mutation (CRT-delta LIR), bound His-LC3 (Fig. 5D). This interaction was strengthened in a dose-dependent manner by TG treatment (Fig. 5E). Functionally, unlike wild-type CRT, CRT-delta LIR failed to promote LC3B-I to LC3B-II conversion (Fig. 5F and Supplementary Fig. 2), TG-induced cell motility, or sphere formation (Fig. 5G, H). In addition, under TG treatment, CRT silencing decreased xenograft growth and suppressed EMT- and autophagy-related signaling, which were recovered by wild-type CRT but not by the LIR mutant (Fig. 5i, j and Supplementary Fig. 3). These data support a role for the CRT-LIR motif in LC3B binding and stress-associated autophagy-related phenotypes, although the precise subcellular topology underlying this interaction remains to be defined.

### GATA6 transcriptionally activates CRT and contributes to the CRT-autophagy axis

**Fig. 6:**
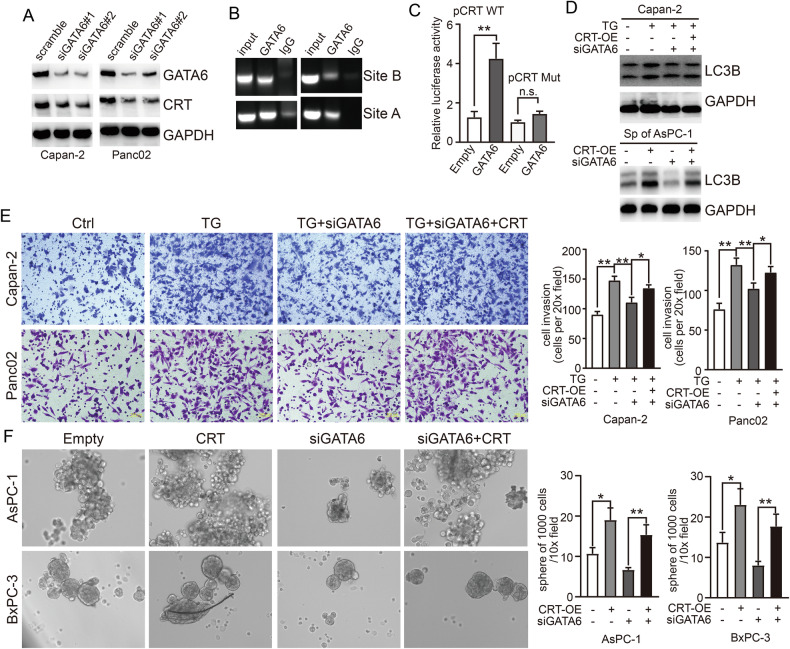
GATA6 directly activates CRT and cooperates with CRT to regulate autophagy-associated phenotypes. **A** Western blotting showing that GATA6 silencing significantly downregulates CRT protein expression in Capan-2 and Panc02 cells. **B** ChIP assays confirming GATA6 binding to the CRT promoter in Capan-2 and Panc02 cells. **C** Luciferase reporter assay in HEK293T cells co-transfected with wild-type (CRT-WT) or mutant (CRT-Mut) CRT promoter plasmids and either a GATA6 overexpression plasmid or empty vector. **D** LC3B expression analyzed by western blotting in Ctrl, TG, TG plus siGATA6, and TG plus siGATA6 combined with CRT-OE groups. **E** Cell invasion under the treatments described in (**D**). **F** Sphere numbers in Ctrl, CRT-OE, siGATA6, and siGATA6 plus CRT-OE groups. Error bars represent SD. *P < 0.05; **P < 0.01 compared with control.

To identify upstream regulators of CRT, we integrated transcription factor prediction algorithms with TCGA co-expression data, nominating GATA6 and ZBTB26 as top candidates (Supplementary Fig. 4a–c). However, GATA6 knockdown, but not ZBTB26 knockdown, significantly reduced both CRT protein and mRNA expression (Fig. 6A and Supplementary Figs. 2 and 4d, e). Predicted GATA6 binding sites within the CRT promoter region from the JASPAR database, together with primers designed for the potential binding sites, are shown in Supplementary Fig. 4f. ChIP assays (Fig. 6B) and luciferase reporter assays (Fig. 6C) validated that GATA6 directly binds the CRT promoter at two specific sites and drives its transcription. Functionally, GATA6 and CRT cooperatively regulated autophagy and malignant phenotypes: GATA6 knockdown reduced autophagy, invasion, and sphere formation in vitro, whereas CRT overexpression significantly reversed these effects (Fig. 6D–F). Thus, GATA6 acts as a direct transcriptional activator of CRT and contributes functionally to this pro-tumorigenic regulatory axis.

### CRT-associated autophagy contributes to metastasis and tumor growth in vivo

**Fig. 7:**
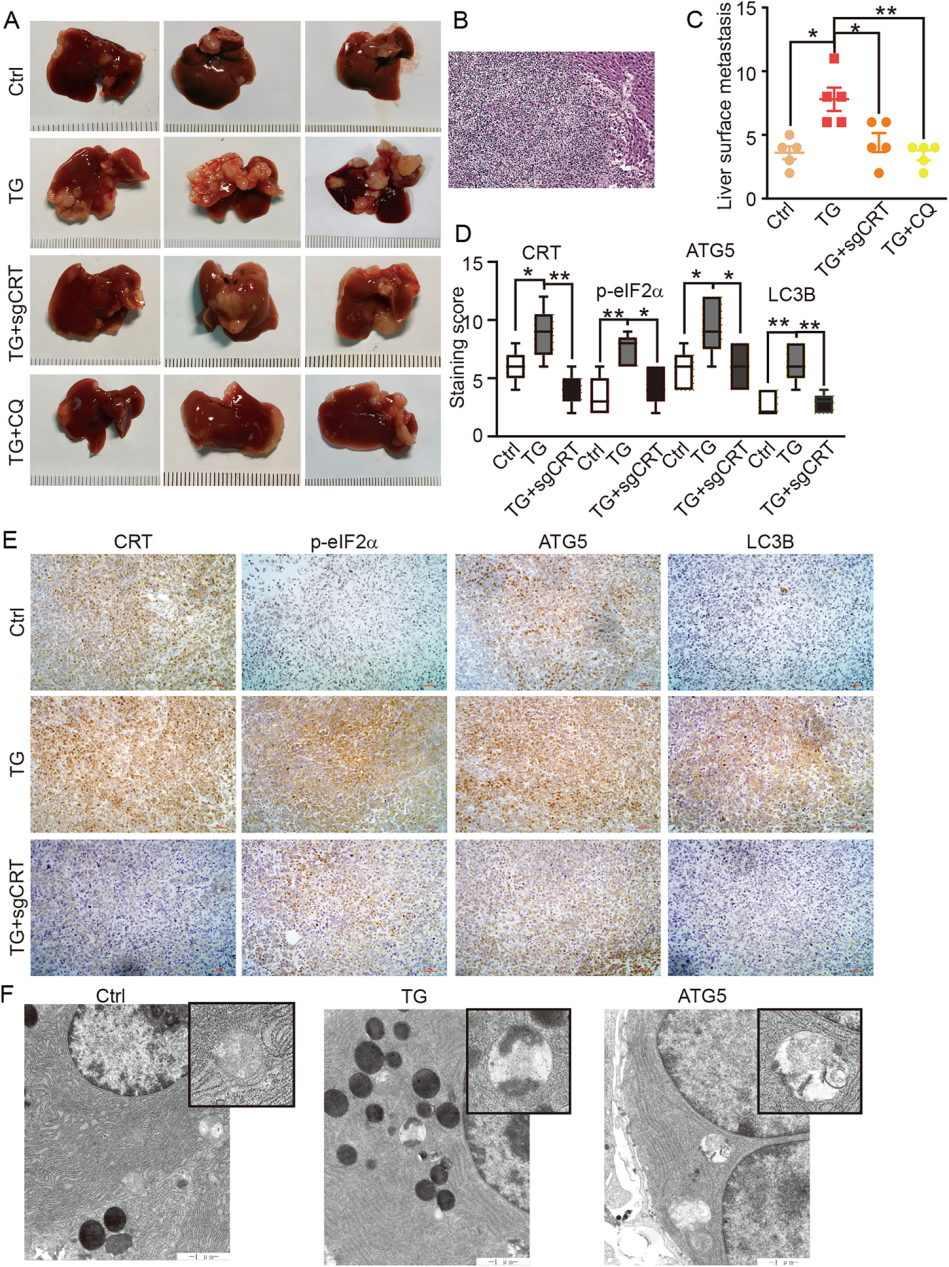
CRT promotes TG-induced distant liver metastasis in vivo. **A** Representative macroscopic images of livers exhibiting metastatic nodules across different treatment groups. **B** H&E staining of liver sections confirming metastatic lesions. **C** Quantification of liver metastatic foci in Ctrl, TG, TG + sgCRT1, and TG + CQ groups. **D** Quantification and representative IHC images for CRT, p-eIF2α, ATG5, and LC3B. **E** Representative IHC images showing CRT, p-eIF2α, ATG5, and LC3B expression in liver metastases from Ctrl, TG, and TG + sgCRT groups. **F** TEM visualization of autophagic structures and ER morphology. Blue arrows indicate autophagosomes; white arrows indicate normal ER; red arrows indicate disorganized ER. Error bars represent SD. *P < 0.05; **P < 0.01 compared with control.

In an immunocompetent liver metastasis model, TG treatment significantly increased metastatic burden, which was suppressed by CRT knockdown or CQ (Fig. 7A–C). IHC analysis of metastases showed that TG upregulated CRT, p-eIF2α, ATG5, and LC3B, and these effects were reversed by CRT silencing (Fig. 7D, E). TEM further showed that TG-induced ER disorganization and autophagosome accumulation were mitigated by CRT knockdown (Fig. 7F).

**Fig. 8:**
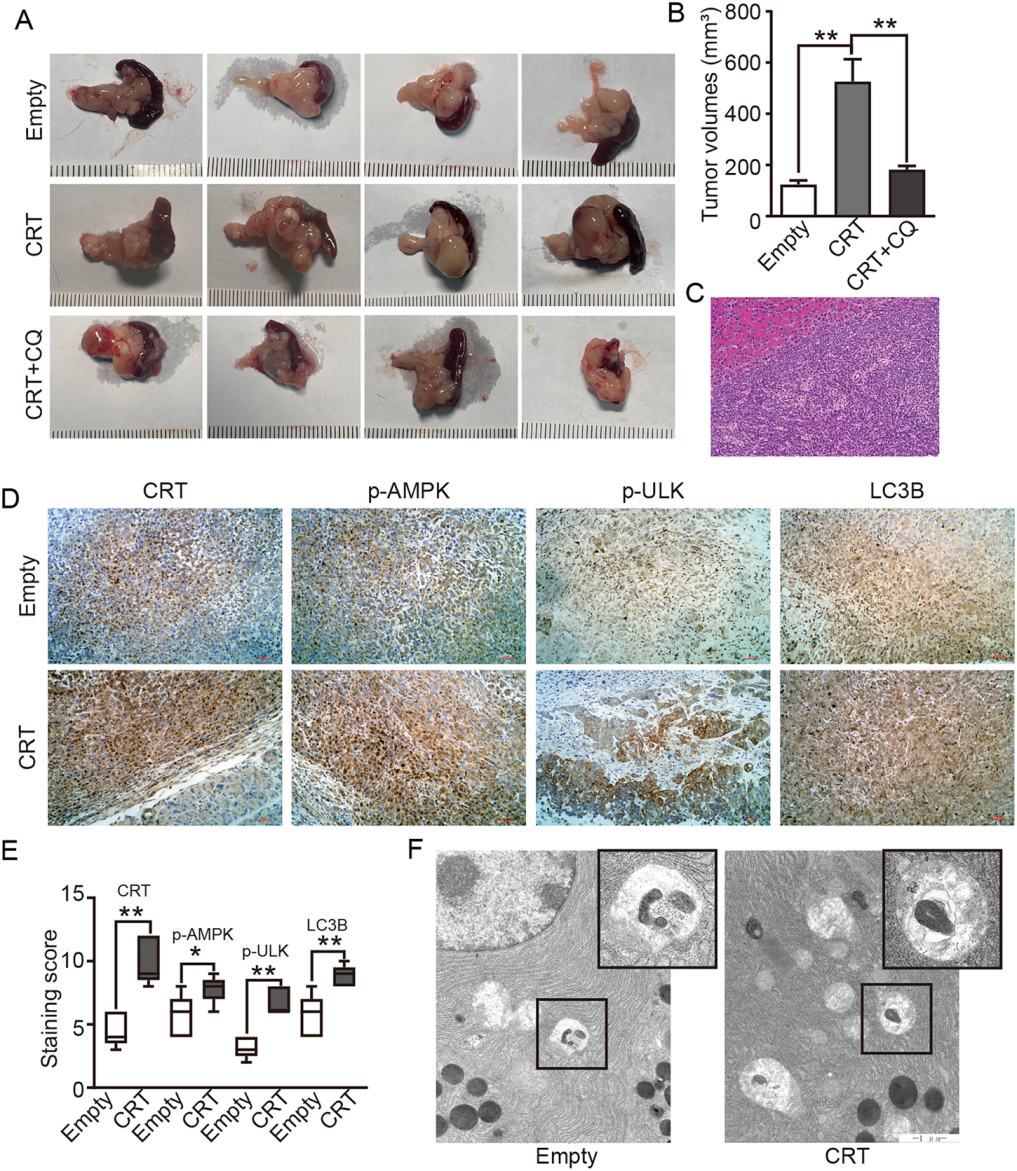
CRT promotes orthotopic pancreatic tumor growth in vivo. **A** Representative images of orthotopic pancreatic tumors from Ctrl, CRT-OE, and CRT-OE plus CQ groups. **B** Quantification of orthotopic pancreatic tumor size or weight across the experimental groups. **C** Histopathological examination of tumor tissues by H&E staining. Quantification (**D**) and representative IHC images (**E**) showing expression of CRT, p-AMPK, p-ULK1, and LC3B in tumors from Ctrl, CRT-OE, and CRT-OE + CQ groups. **F** TEM visualization of autophagosomes in Ctrl and CRT-OE groups. Blue arrows indicate autophagosomes. Error bars represent SD. *P < 0.05; **P < 0.01 compared with control. CQ chloroquine.

In an orthotopic model established from sphere-derived AsPC-1 cells, CRT overexpression accelerated primary tumor growth, whereas CQ treatment reversed this phenotype, supporting its autophagy dependence (Fig. 8A, B). H&E staining, IHC, and TEM analyses confirmed concurrent upregulation of the CRT/autophagy pathway in these tumors (Fig. 8C–F). These in vivo studies further support a role for CRT-driven autophagy in promoting local tumor progression and distant metastasis.

### Clinical associations of the GATA6-CRT-autophagy network in human PC

**Fig. 9:**
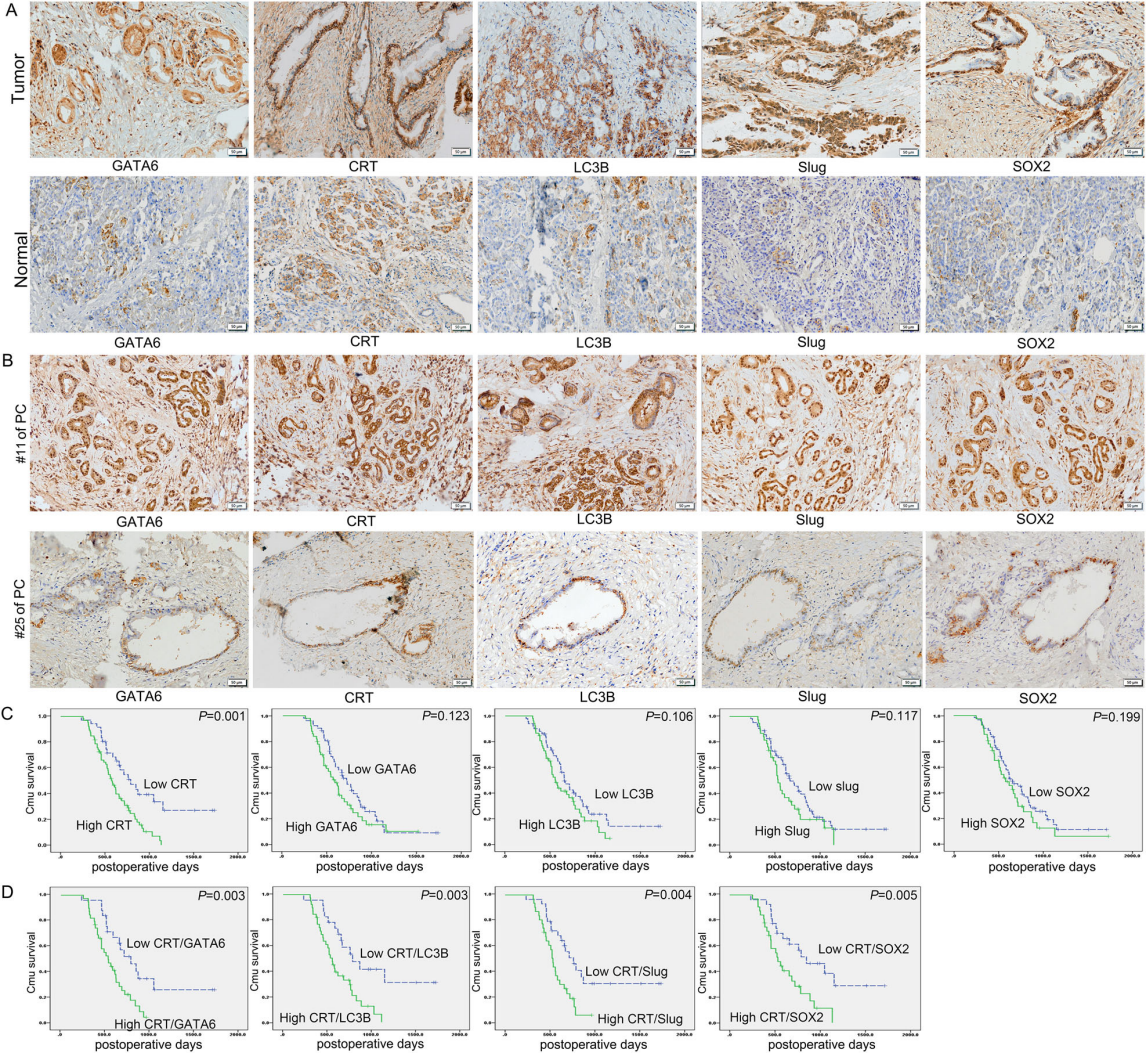
Association between key protein expression and clinical outcomes in human PC specimens. **A** Representative IHC images showing GATA6, CRT, LC3B, Slug, and SOX2 expression in human pancreatic cancer tissues and adjacent normal pancreatic tissues. **B** IHC staining showing high (specimen #11) and low (specimen #25) expression levels of the indicated proteins in two representative PC cases. **C** Kaplan–Meier survival analysis comparing overall survival between patients with high versus low expression of the indicated proteins. **D** Kaplan–Meier curves evaluating overall survival based on co-expression patterns of CRT with GATA6, LC3B, Slug, or SOX2. Error bars represent SD. *P < 0.05; **P < 0.01 compared with control.

Finally, we assessed the clinical relevance of our findings in human PC specimens. GATA6, CRT, LC3B, Slug, and SOX2 were significantly overexpressed in tumors compared with adjacent normal tissues (Fig. 9A, B). CRT expression positively correlated with GATA6, LC3B, Slug, and SOX2 (Table 1). Serial sections demonstrated co-expression of these markers in individual patient samples (Fig. 9B). The association of CRT with these proteins in PDAC was further verified using TCGA data (Supplementary Fig. 5). Although high CRT expression alone predicted poor survival, co-expression of CRT with GATA6, LC3B, Slug, or SOX2 identified patient subgroups with the worst prognosis (Fig. 9C, D). Cox proportional hazards analyses showed that CRT expression remained an independent unfavorable prognostic factor (Table 2). These clinical data support the clinical relevance of this network, while remaining correlative rather than definitive mechanistic validation.

**Table 1:** The relationship of CRT with GATA6 and the critical markers of ERS and autophagy signaling.

Parameters		CRT		Spearman ranks	*P*
		Low (36)	High (66)		
GATA6	Low (52)	25	27	0.273	0.006
	High (50)	11	39		
LC3B	Low (50)	23	27	0.220	0.027
	High (52)	13	39		
Slug	Low (64)	29	35	0.272	0.006
	High (38)	7	31		
SOX2	Low (61)	27	34	0.229	0.021
	High (41)	9	32

**Table 2:** Survival data in univariate and multivariate analysis.

Parameters	Median survival (days)	Univariate analysis *P* (log rank)	Multivariate analysis hazard ratio (95% CI)	*P*
Age (<65/≥65 years)	658/620	0.451	─	
Gender (male/female)	618/684	0.511	─	
Tumor location (Head/Body-tail)	657/536	0.116	─	
Tumor size (<3/≥3 cm)	765/586	0.133	─	
Well/poor/moderate Differentiation	758/715/506	0.004	1.453(1.026–2.058)	0.035
T stage (T1 + T2/ T3)	655/620	0.282	─	
Lymph nodes metastasis (N0/N1)	668/586	0.089	─	
TNM stage(I + II/III)	668/455	0.001	2.251(1.192–4.249)	0.012
Vascular permeation (absent/present)	657/556	0.024	1.497(0.923–2.428)	0.102
CA19-9 level (<137 U/ml/≥37 U/ml)	765/606	0.147	─	
CRT (high/low)	786/571	0.001	1.869(1.079–3.239)	0.026
GATA6 (high/low)	710/580	0.123	─	
LC3B (high/low)	658/560	0.106	─	
SOX2(high/low)	657/580	0.199	─	
Slug(high/low)	668/534	0.117	─	

## Discussion

This study identifies a previously unappreciated GATA6-CRT regulatory axis associated with ERS, autophagy-related responses, and phenotypic plasticity in PC. Our data indicate that CRT, transcriptionally regulated by GATA6, contributes to EMT-associated and stemness-associated phenotypes under distinct stress conditions. Under proteotoxic stress, CRT was linked to PERK/eIF2α-associated autophagy signaling and EMT-related changes, whereas under nutrient stress, it was associated with AMPK/mTOR/ULK1 signaling and sphere-forming, stemness-associated traits. We therefore interpret CRT as an important stress-responsive regulator in the experimental contexts examined here, rather than as a universal master regulator across PDAC.

Although autophagy has been independently implicated in EMT and CSC maintenance [12–14, 26–28], the molecular factors that connect these processes in PDAC remain incompletely defined. Our findings support a model in which CRT links ER proteostasis to autophagy-associated EMT and stemness-related phenotypes. At the same time, our data do not fully delineate every step of autophagic flux regulation; therefore, our conclusions focus on autophagy-associated phenotypes under stress rather than a fully resolved autophagic mechanism.

Our data from serum-free cultures suggest that CRT contributes to the cellular response to nutrient deprivation and is associated with AMPK/mTOR/ULK1 signaling, sphere formation, and expression of stemness-related markers. The in vivo findings in Kras^G12D^ models and transplantation assays further support a role for CRT in tumor-initiating potential and stress-adaptive phenotypes. However, these observations are most appropriately interpreted as evidence for stemness-associated traits and tumor-initiating capacity, rather than as definitive demonstration of all functional properties of CSCs.

A notable finding of our study is the evidence that CRT interacts with LC3 through a conserved LIR motif. The Co-IP, co-localization, GST pull-down, and CRT-delta LIR rescue data support the functional importance of this region in stress-associated autophagy-related phenotypes. Nevertheless, an important cell-biological question remains unresolved: CRT is canonically an ER luminal chaperone, whereas LC3 is cytosolic. Accordingly, although our data support an interaction, they do not yet fully explain the subcellular topology or intermediary steps that permit this association in cells. One plausible scenario is that under severe ER stress, CRT may access the cytosol through ER membrane permeabilization or during ribosome stalling/translational pausing; alternatively, the interaction may occur at ER-phagy receptor contact sites. This issue warrants explicit caution and should be addressed in future studies.

Upstream, we identify GATA6 as a direct transcriptional activator of CRT. This finding places the CRT-autophagy program within a lineage-associated transcriptional context and suggests one mechanism by which stress adaptation may be coupled to tumor cell state. Given the context-dependent roles of GATA6 in pancreatic and other cancers [29–34], we interpret this result as defining a GATA6-dependent regulatory input into CRT expression under the conditions studied here, rather than as establishing a universally dominant role for GATA6 in PDAC biology.

The multigene prognostic signature identified through bioinformatic analyses provided a rationale for focusing on CRT. To further dissect the relative contribution of CRT to the prognostic power of this signature, multivariate Cox regression analysis in both our cohort and the TCGA dataset showed that CALR (CRT) expression retained independent prognostic significance after adjustment for other signature genes. These findings suggest that CRT represents a functionally validated component of the network, while its prognostic value complements, rather than replaces, the contribution of other signature genes. Likewise, the clinical tissue analyses and survival associations support the clinical relevance of the GATA6-CRT-autophagy network, but remain correlative and should not be interpreted as definitive mechanistic validation.

We also acknowledge that the number of scRNA-seq samples analyzed in this study was limited, which may constrain the generalizability of the identified epithelial subprograms. Therefore, although our single-cell analyses revealed dynamic transcriptional states associated with CRT expression, these findings should be considered hypothesis-generating and require validation in larger, independent scRNA-seq cohorts. Ideally, future studies should incorporate longitudinal samples to capture the temporal evolution of these subprograms during disease progression or treatment.

**Fig. 10:**
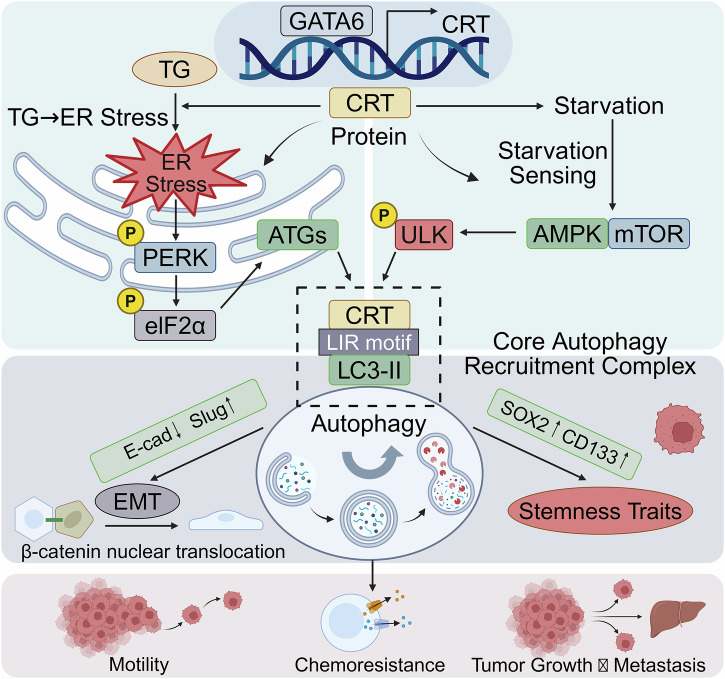
Overall schematic summary of the proposed model. This study supports a model in which TG-induced ERS and serum-free stemness-enriching conditions are associated with autophagic activation in pancreatic cancer. On one hand, CRT promotes ERS-associated EMT-related changes together with activation of the PERK/eIF2α axis and the ATG5/ATG12/LC3B signaling pathway. On the other hand, CRT supports stemness-associated traits in the setting of autophagy linked to the AMPK/mTOR/ULK1 signaling cascade. The functional interaction between CRT and LC3 depends on the LIR motif within CRT, although the precise subcellular topology of this interaction remains to be clarified. GATA6 acts as a direct transcriptional activator of CRT expression. Overall, the GATA6-CRT axis is proposed to contribute to autophagy-associated plasticity, chemoresistance, tumor growth, and metastasis in pancreatic cancer.

In summary, as schematized in Fig. 10, our data support a model in which the GATA6-CRT axis links transcriptional regulation, stress signaling, and autophagy-associated phenotypes to promote EMT-related changes, stemness-associated traits, tumor growth, and metastasis in PC. By integrating cellular, animal, and clinical observations, this study provides a mechanistically informed framework for understanding how stress adaptation may contribute to PDAC aggressiveness. The GATA6-CRT-autophagy network therefore represents a candidate therapeutic vulnerability, although additional work will be required to define its full scope, topology, and context dependence in human disease.

## Supplementary information

Supplementary Figure and Table Legends.docx

Supplemental Table 1

Supplementary Table 2

Supplemental Figure 1

Supplemental Figure 2

Supplemental Figure 3

Supplemental Figure 4

Supplemental Figure 5

Original Data of WB

Original Data of PCR

## Data Availability

Materials generated in this study are available from the corresponding author upon reasonable request. Publicly available datasets analyzed in this study are described in the Methods section and cited accordingly, including the previously published dataset associated with 10.7150/ijbs.102381 [20].
