# Supplementary material for: GATA6-CRT axis promotes stress-associated autophagy, EMT, and stemness-associated traits in pancreatic cancer
Source: Cell Death Dis. 2026 Jun 4;17(1):610. doi: 10.1038/s41419-026-08914-8 (PMC13323382; doi:10.1038/s41419-026-08914-8)
Supplement: Supplementary file 1 — Supplementary Figure and Table Legends.docx [file 41419_2026_8914_MOESM1_ESM.docx]

**Supplementary Figure Legends**

Supplementary Figure 1. CRT silencing inhibits glucose insufficiency-induced ER stress and autophagy in vitro. A. Western blotting and quantification of indicated proteins in Ctrl, 4-PBA, LM, and 4-PBA plus LM groups. B. Western blotting and quantification of indicated proteins in Ctrl, LM, LM plus sgCRT1, and LM plus CQ groups. C. Cell invasion in Ctrl, LM, LM plus sgCRT1, LM plus 4-PBA, and LM plus CQ groups. LM: low-glucose medium; CQ: chloroquine. Error bars represent SD. *P < 0.05; **P < 0.01 compared with control.

Supplementary Figure 2. Quantitative analysis of western blot results shown in Fig. 4C-E, Fig. 5F, Fig. 6A, and Fig. 6D.

Supplementary Figure 3. Representative IHC images of E-cadherin, Slug, and LC3B expression in scramble, sgCRT, sgCRT + CRT-ΔLIR, and sgCRT + CRT-OE groups under TG treatment.

Supplementary Figure 4. GATA6 is identified as a potential transcriptional activator of CRT. A. Identification of the top nine transcription factors from the intersection of two prediction sets (PWM_Enrich JASPAR and FIMO JASPAR) and CRT co-expressed genes in TCGA. B. GEPIA correlation analysis of CRT with the top nine selected genes based on TCGA. C. TCGA data showing expression of the remaining six genes in pancreatic cancer tissues. D. mRNA levels of ZBTB26 and CRT in si1-ZBTB26 and si2-ZBTB26 groups of Capan-2 cells. E. mRNA levels of GATA6 and CRT in si1-GATA6 and si2-GATA6 groups in Capan-2 and Panc02 cells. F. Predicted GATA6 binding sites within the CRT promoter region from the JASPAR database and primers designed for the potential binding sites.

Supplementary Figure 5. Correlation analysis using GEPIA showing close associations of CRT with GATA6, LC3B, SOX2, and Snail2/Slug in TCGA.

**Supplementary Table Legends**

Supplemental Table 1. The target sequences of sg-CRT1, sg-CRT2, GATA6 and scramble.

Supplementary Table 2. Primers used in this study.
