# Supplementary material for: GATA6-CRT axis promotes stress-associated autophagy, EMT, and stemness-associated traits in pancreatic cancer
Source: Cell Death Dis. 2026 Jun 4;17(1):610. doi: 10.1038/s41419-026-08914-8 (PMC13323382; doi:10.1038/s41419-026-08914-8)
Supplement: Supplementary file 2 — Supplemental Table 1 [file 41419_2026_8914_MOESM2_ESM.docx]

Supplemental Table 1. The target sequences of sg-CRT1, sg-CRT2, GATA6 and scramble.

| Gene | Oligo Name | Oligo Sequence |
| --- | --- | --- |
| CRT-homo | sg-CRT1 | GAAGATGACATGAACCTTCT |
|  | sg-CRT2 | CGAGCCTGCCGTCTACTTCA |
| CRT-mus | sg-CRT1 | GAGCGGCACCGAAAGGAGCA |
| sgRNA | Scramble | TTCTCCGAACGTGTCACGT |
| GATA6 | siRNA1 | CUCUGGUAAUAGCAAUAAUTT |
| GATA6 | siRNA2 | GCUCAAGUAUUCGGGUCAATT |
| ZBTB26 | siRNA1 | GGUCCUGGAUGCAGGUAAACUTT |
| ZBTB26 | siRNA2 | CAACAGUCACAGACUCUAAAGTT |
| ATG5 | siRNA | CCAUCAAUCGGAAACUCAUTT |
| NC | siCtrl | UUCUCCGAACGUGUCACGUTT |
