# Supplementary material for: GATA6-CRT axis promotes stress-associated autophagy, EMT, and stemness-associated traits in pancreatic cancer
Source: Cell Death Dis. 2026 Jun 4;17(1):610. doi: 10.1038/s41419-026-08914-8 (PMC13323382; doi:10.1038/s41419-026-08914-8)
Supplement: Supplementary file 3 — Supplementary Table 2 [file 41419_2026_8914_MOESM3_ESM.docx]

**Supplementary Table 2.** Primers used in this study

| **Primer name** | **Sequence (5'-3')** |
| --- | --- |
| Homo-CRT-F | GGATCCACCCAGAAAATTGAC |
| Homo-CRT-R | AAACTCCTCAGCGTATGCCT |
| Mouse-CRT-F | AAAGGACCCTGATGCTGCCAAG |
| Mouse-CRT-R | TCAGGGATGTGCTCTGGCTTGT |
| GATA6-F | CCACAACACAACCTACAGCCTCAG |
| GATA6-R | GAGCCCATCTTGACCCGAATACTTG |
| ZBTB26-F | GAGCCACATTGTAGAACGGTGC |
| ZBTB26-R | GGAGAAGCACTCTGTGGTTCAC |
| GAPDH-F | GTCTCCTCTGACTTCAACAGCG |
| GAPDH-R | ACCACCCTGTTGCTGTAGCCAA |
| CRT promotor-F1  (-1545 to -1533) | AATCCGTGAGCTCTCTCC |
|  | CCCATTCCCTCTAGGTGG |
| CRT promotor-F2  (-738 to -726) | CAGAGACCCTTGCCAAGTC |
|  | AGGAAAGAGGGTGGGGAG |
