# Supplementary figures and images for: GATA6-CRT axis promotes stress-associated autophagy, EMT, and stemness-associated traits in pancreatic cancer
Source: Cell Death Dis. 2026 Jun 4;17(1):610. doi: 10.1038/s41419-026-08914-8 (PMC13323382; doi:10.1038/s41419-026-08914-8)

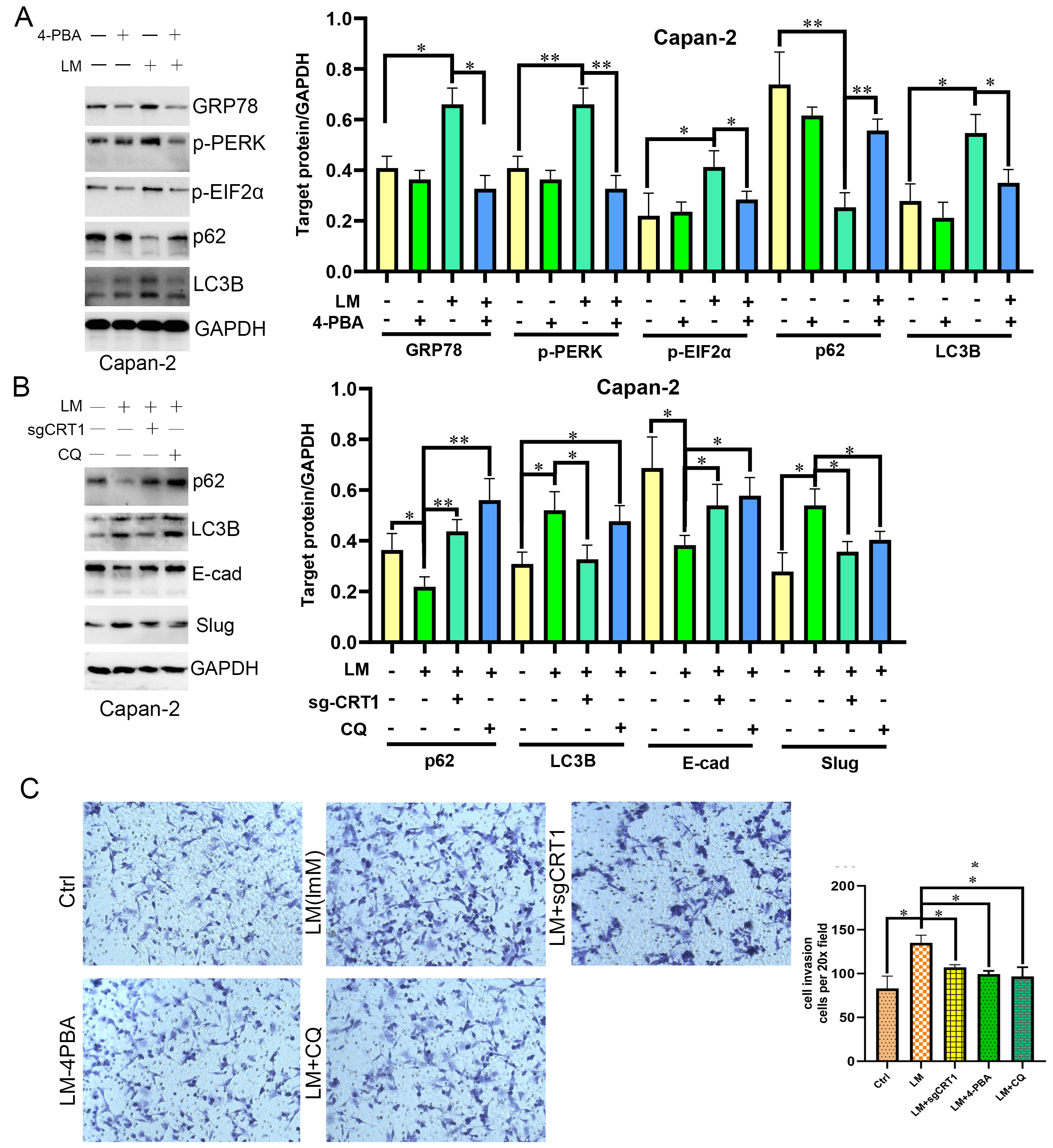

Supplement: Supplementary file 4 — Supplemental Figure 1 [file 41419_2026_8914_MOESM4_ESM.tif]

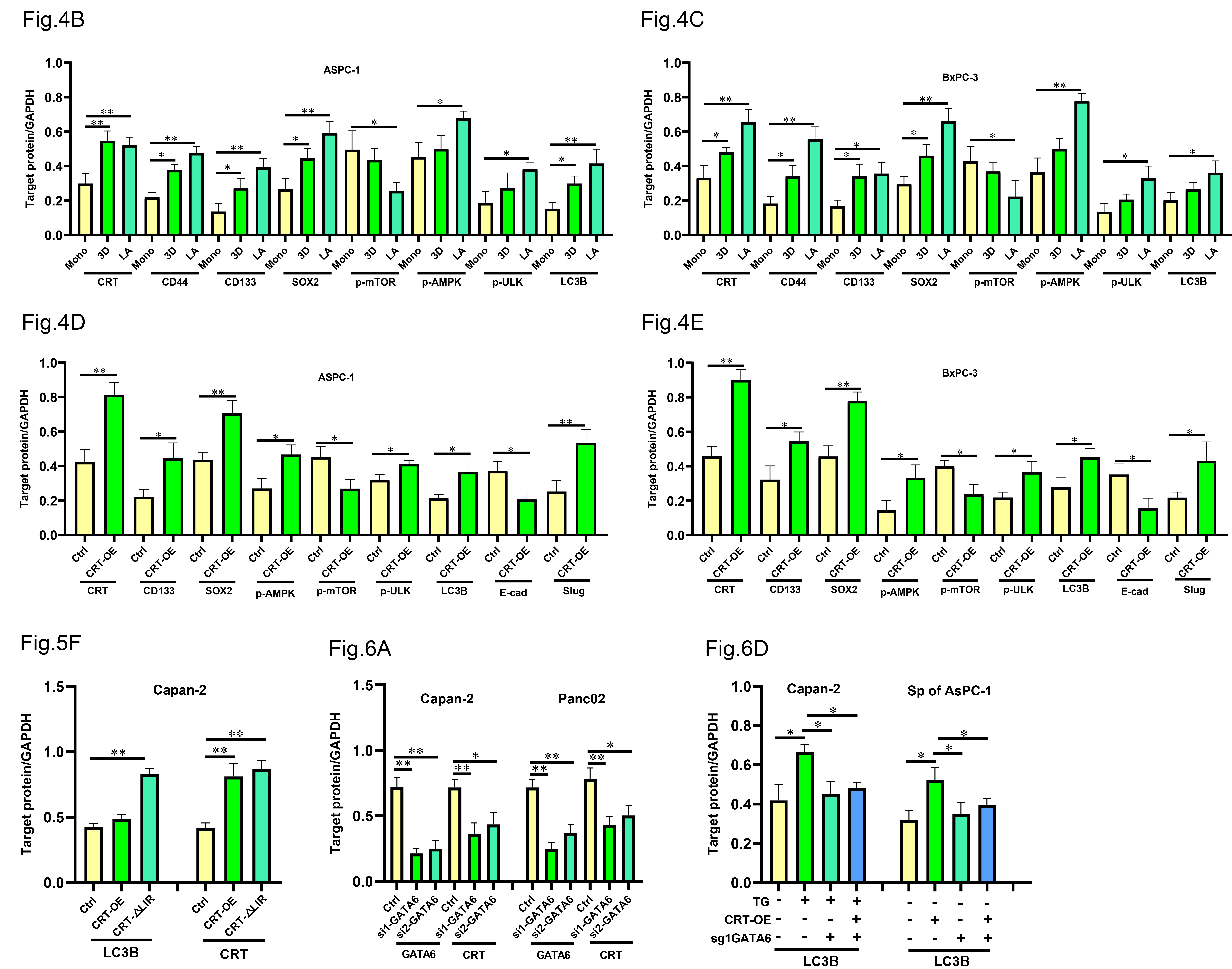

Supplement: Supplementary file 5 — Supplemental Figure 2 [file 41419_2026_8914_MOESM5_ESM.tif]

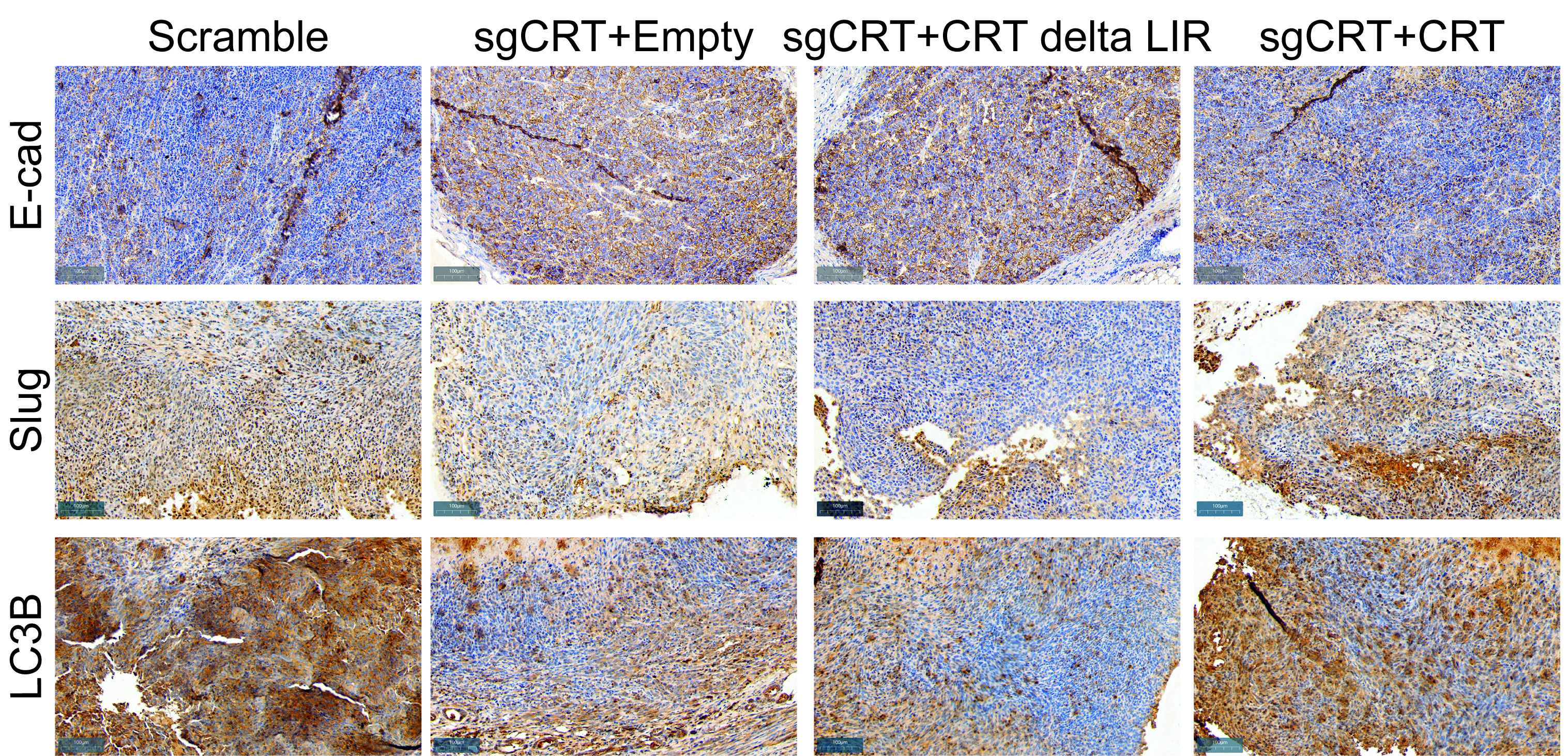

Supplement: Supplementary file 6 — Supplemental Figure 3 [file 41419_2026_8914_MOESM6_ESM.tif]

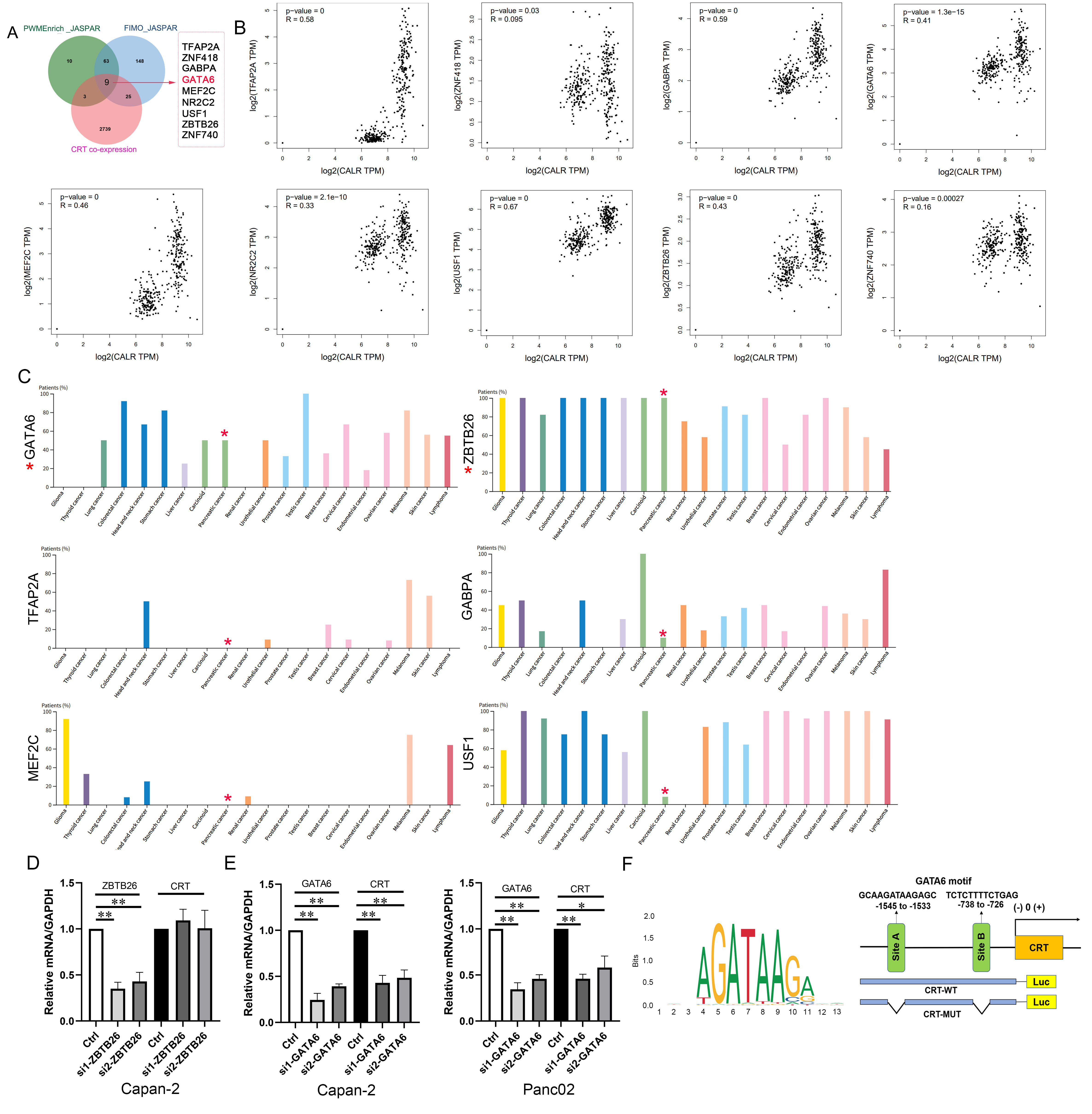

Supplement: Supplementary file 7 — Supplemental Figure 4 [file 41419_2026_8914_MOESM7_ESM.tif]

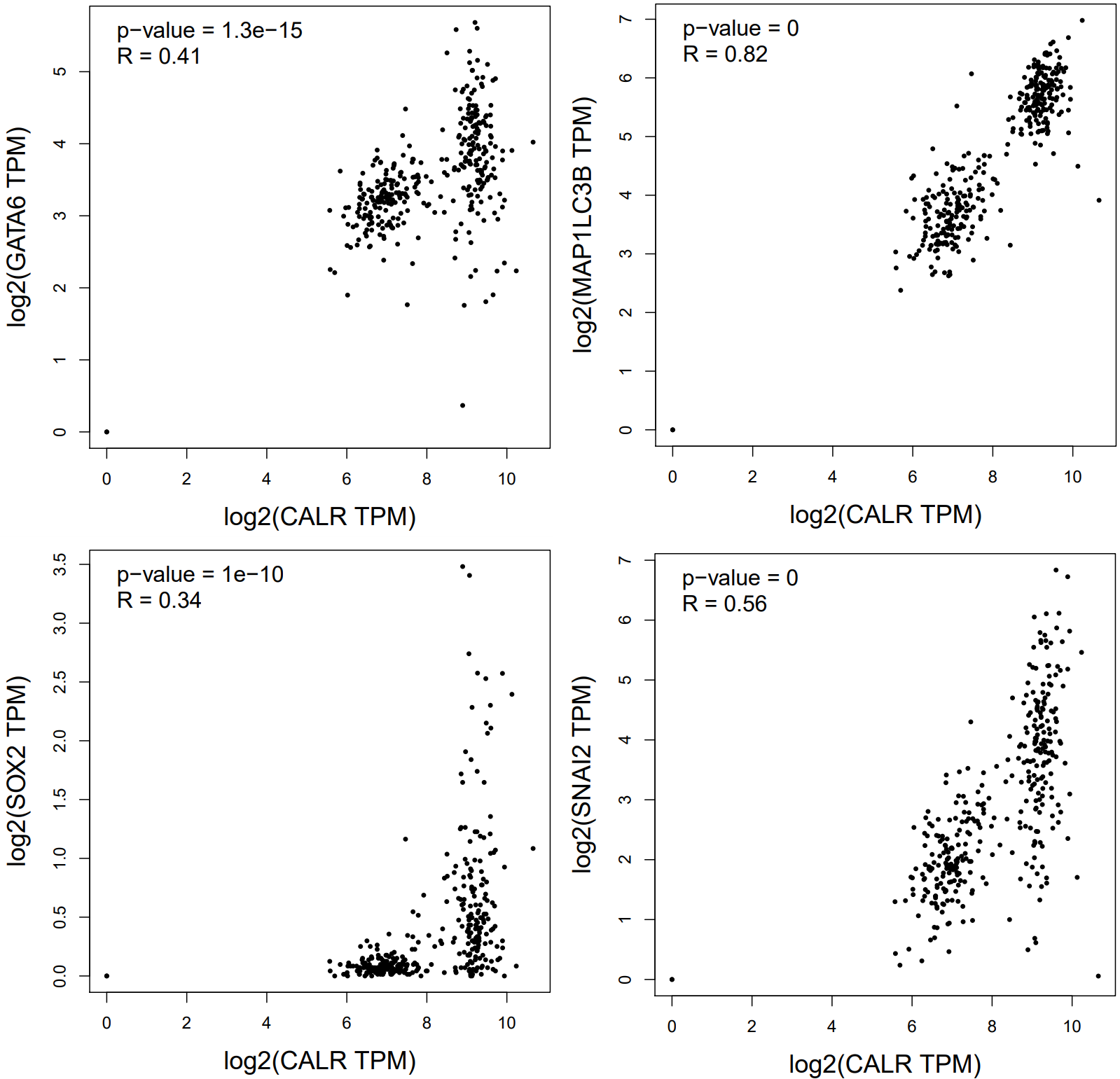

Supplement: Supplementary file 8 — Supplemental Figure 5 [file 41419_2026_8914_MOESM8_ESM.tif]

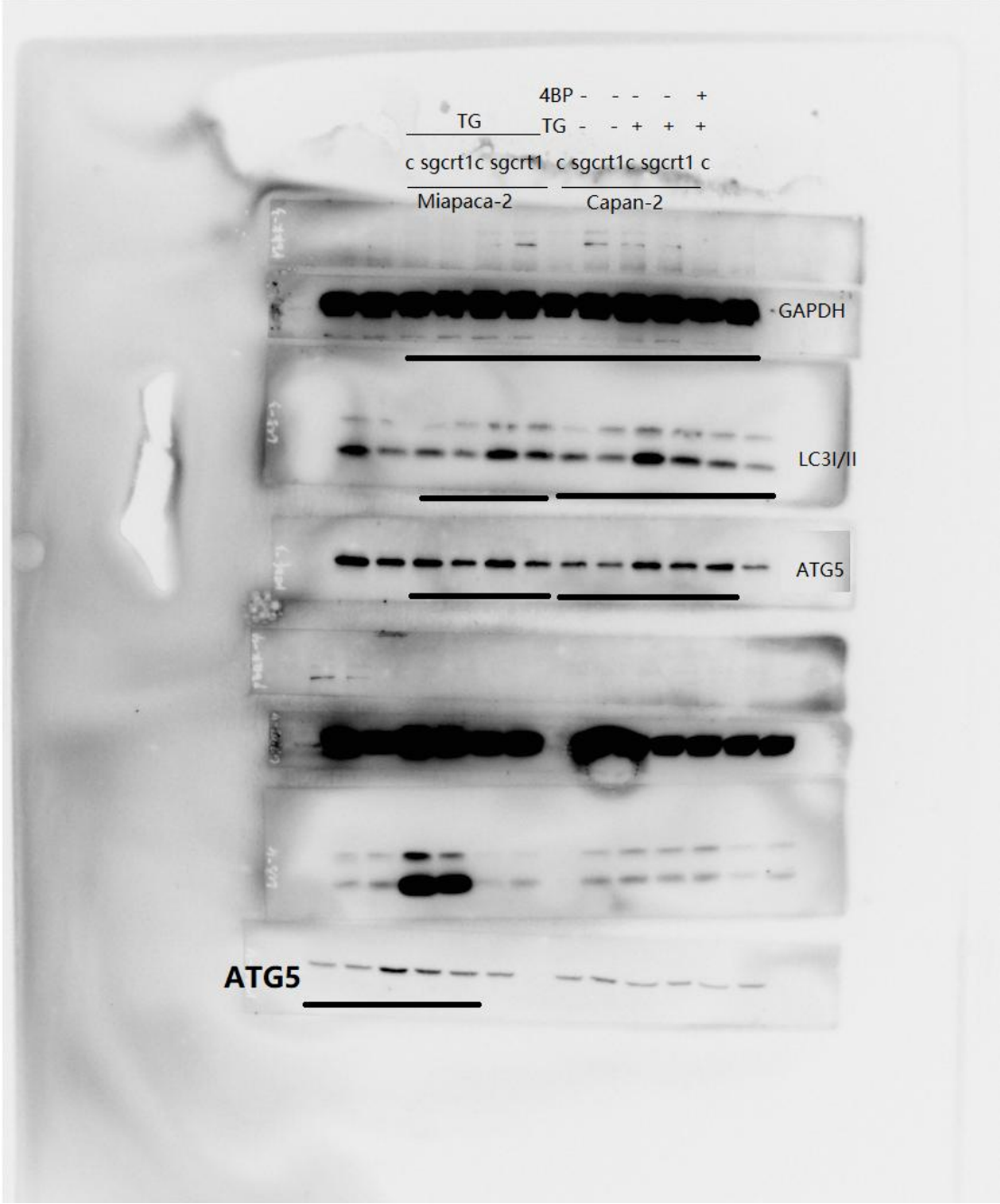

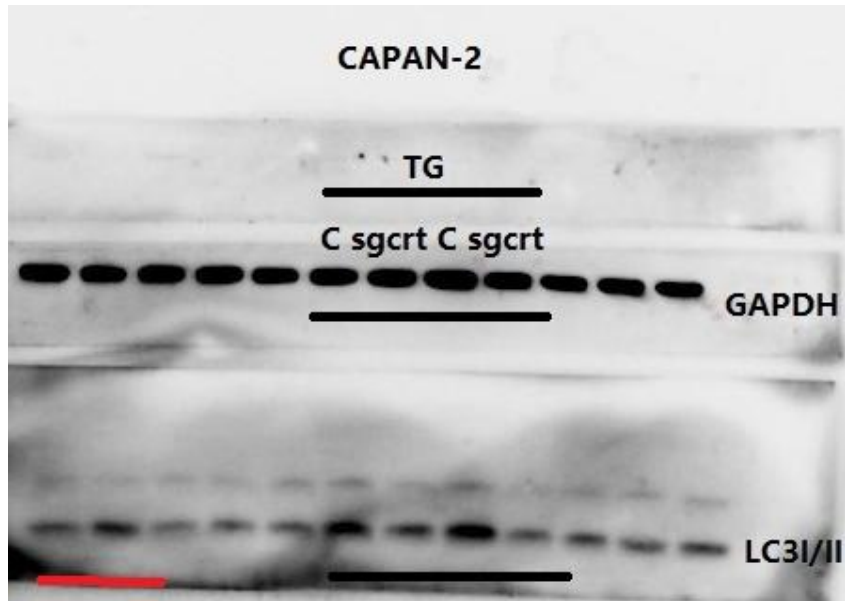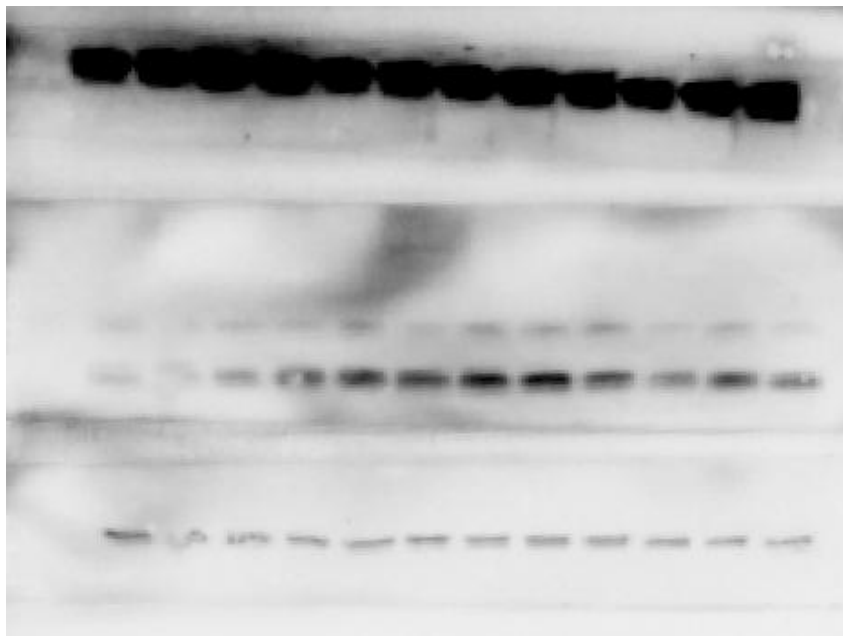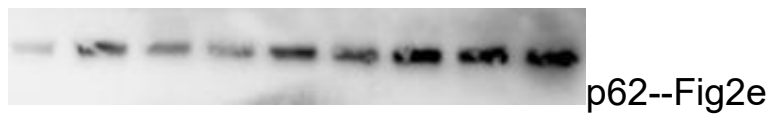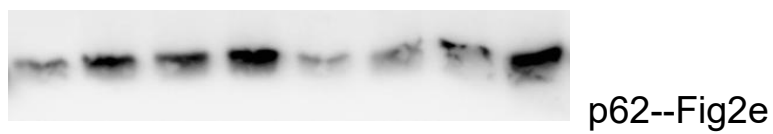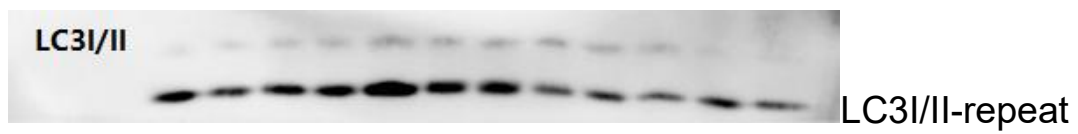



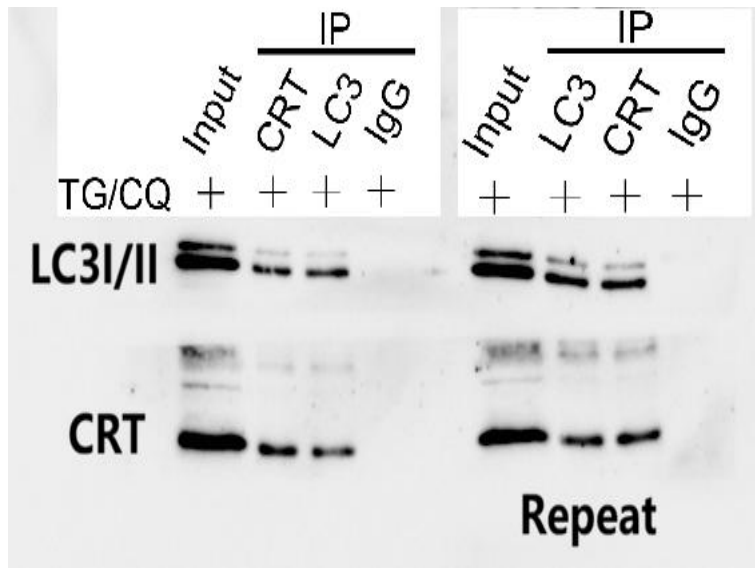

Fig5-1B CO-IP

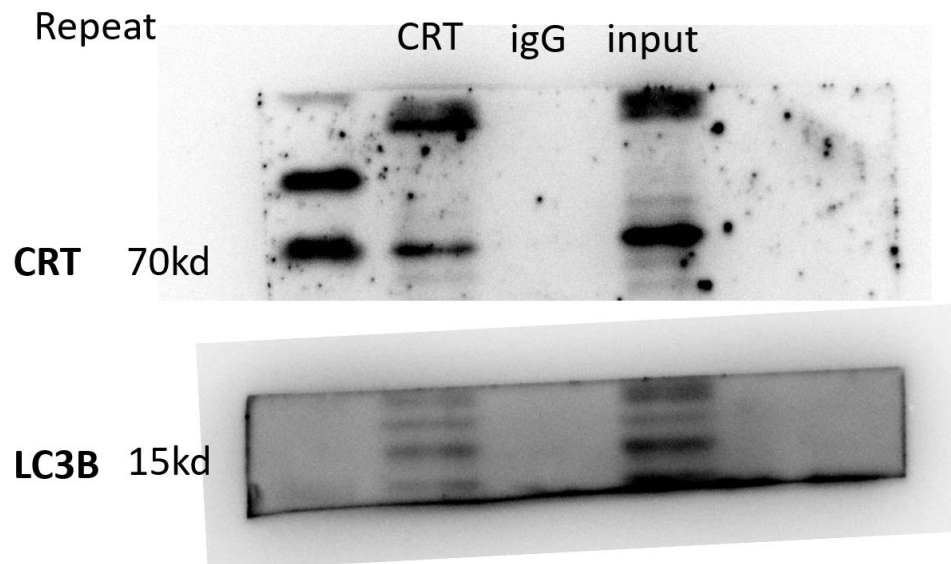

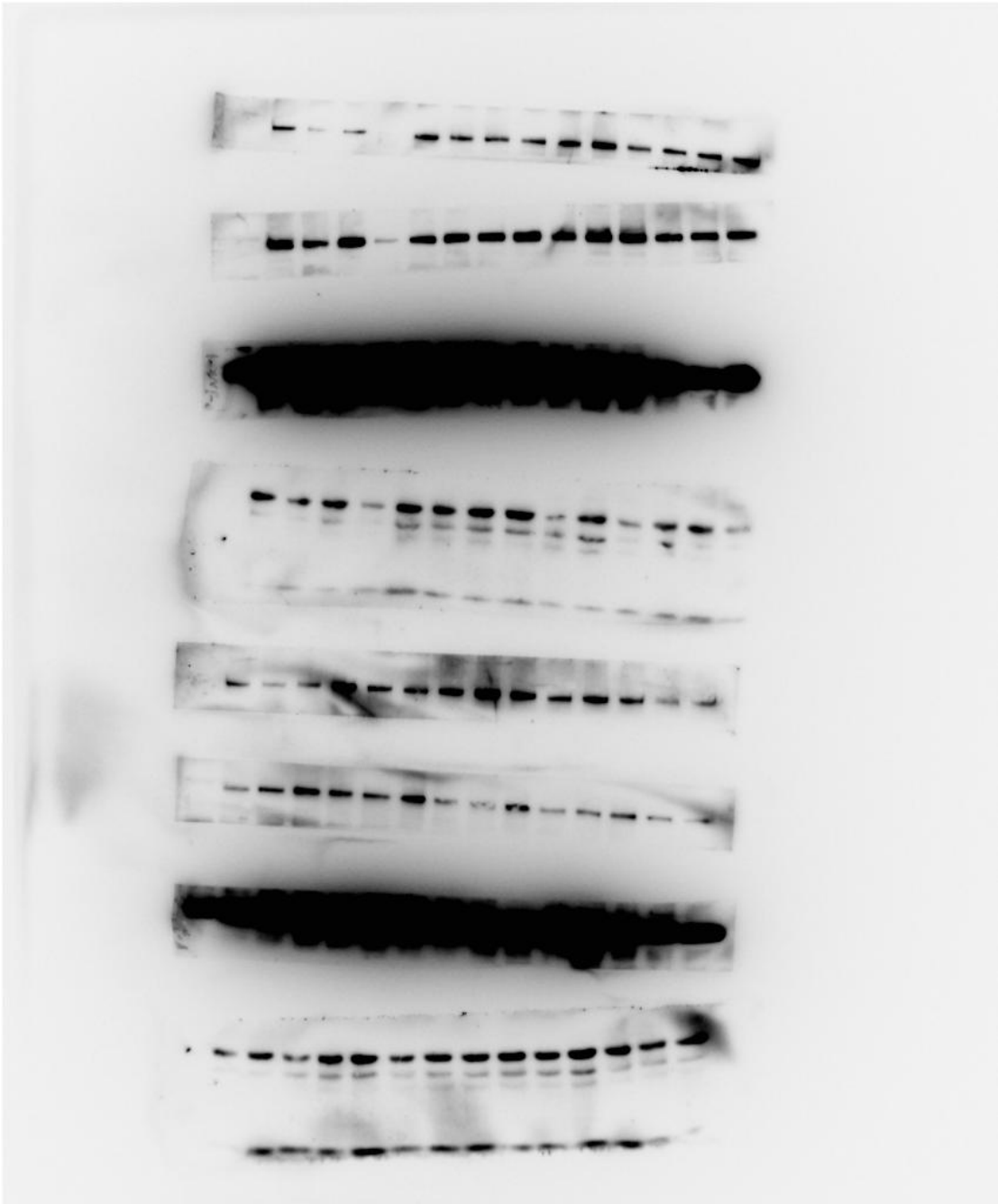

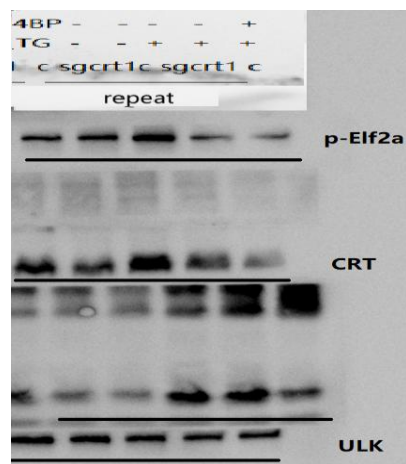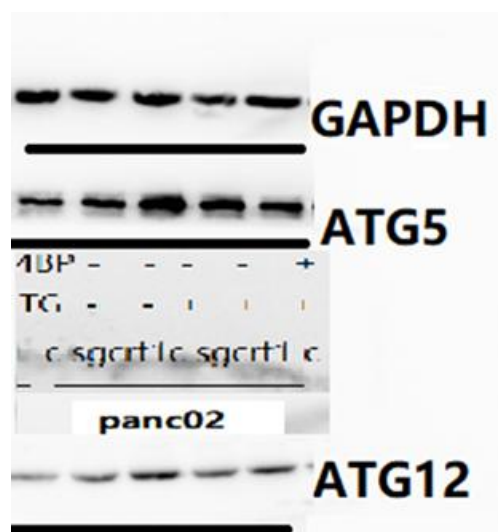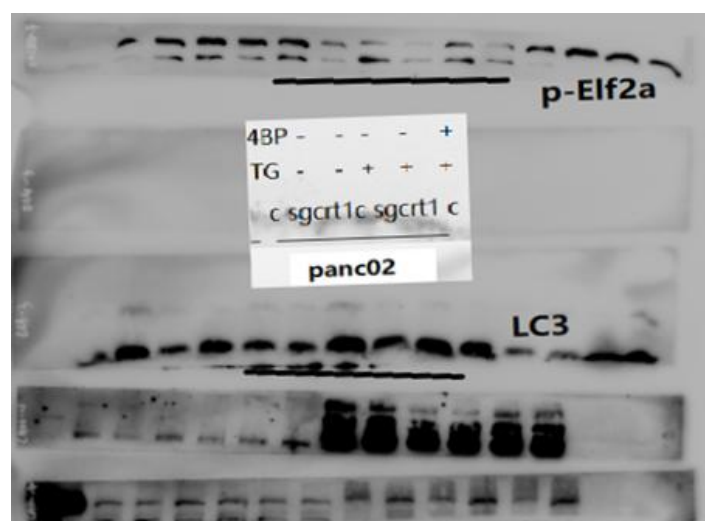

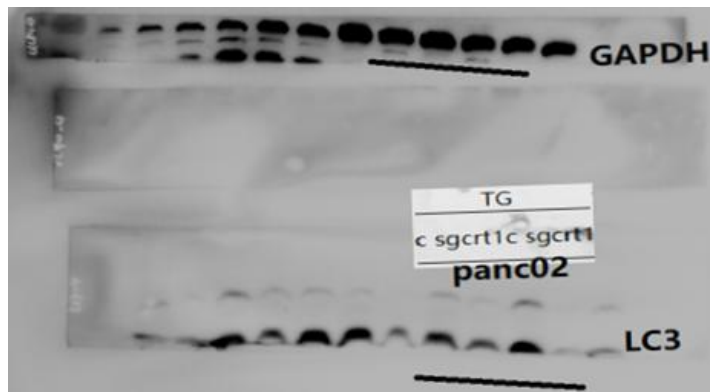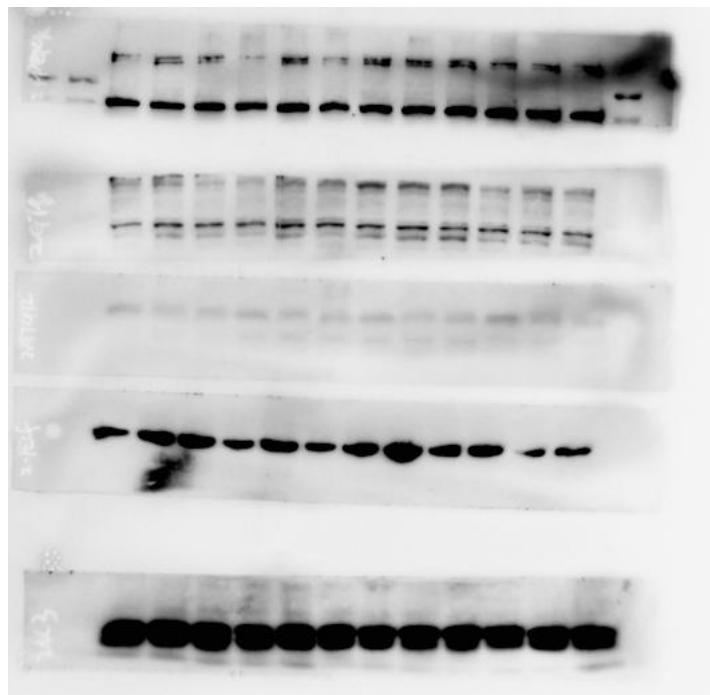

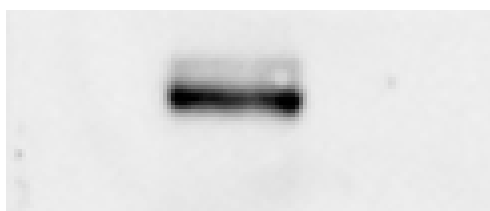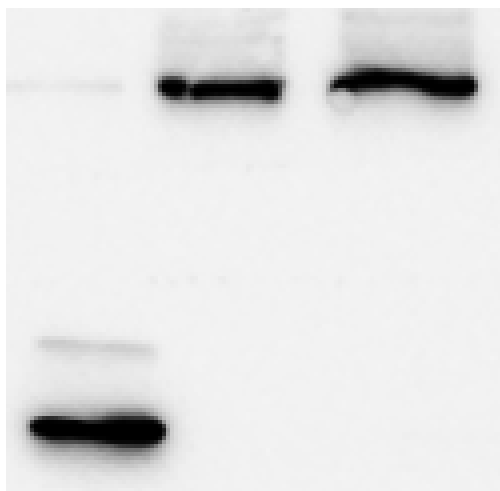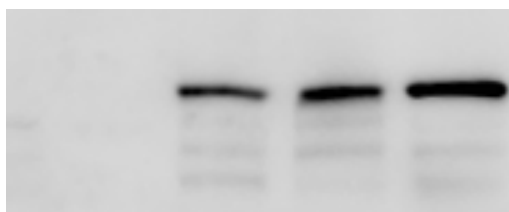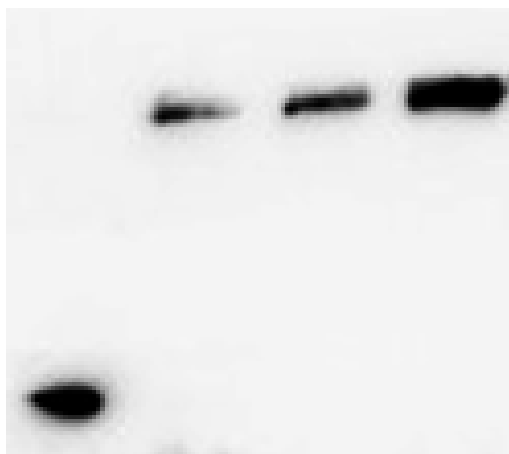

Fig5.D AND E

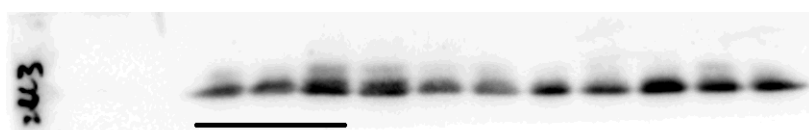

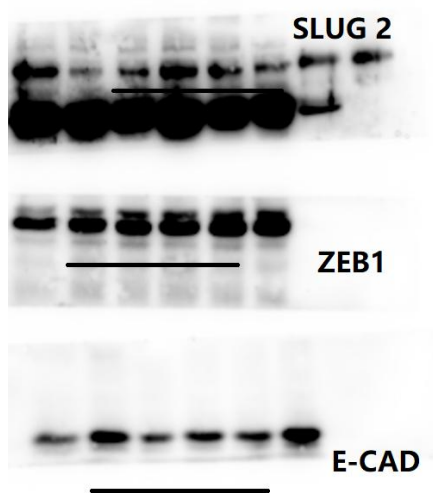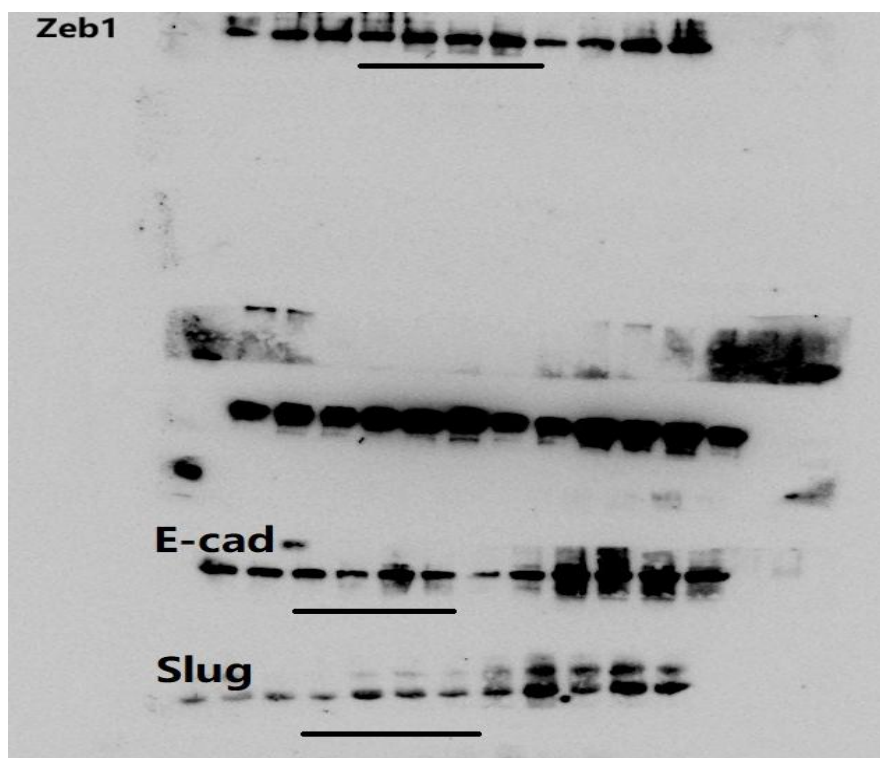

Fig3H

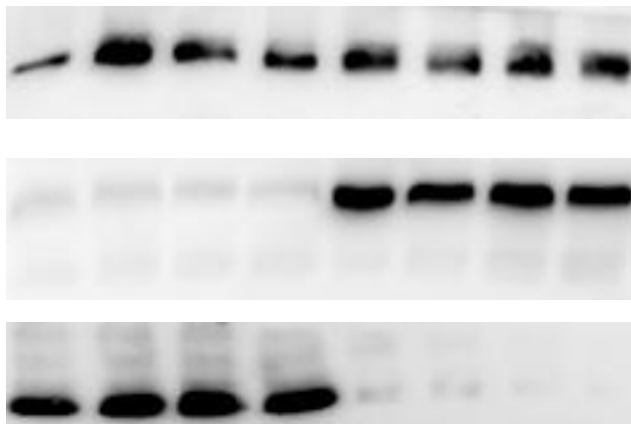

Fig3. J

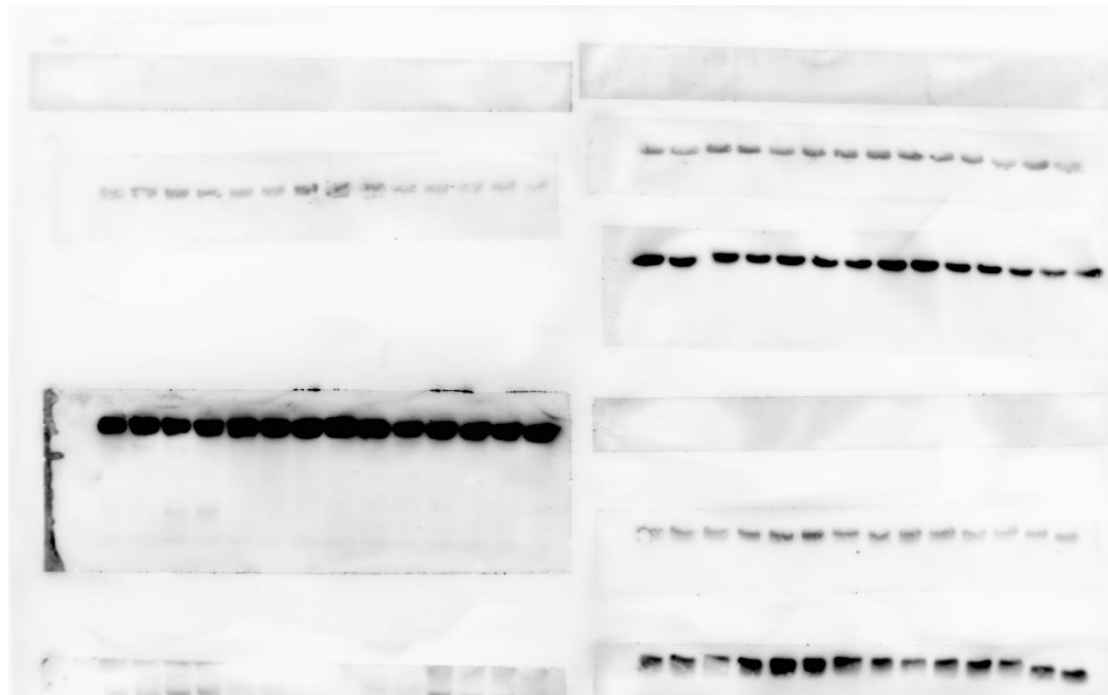

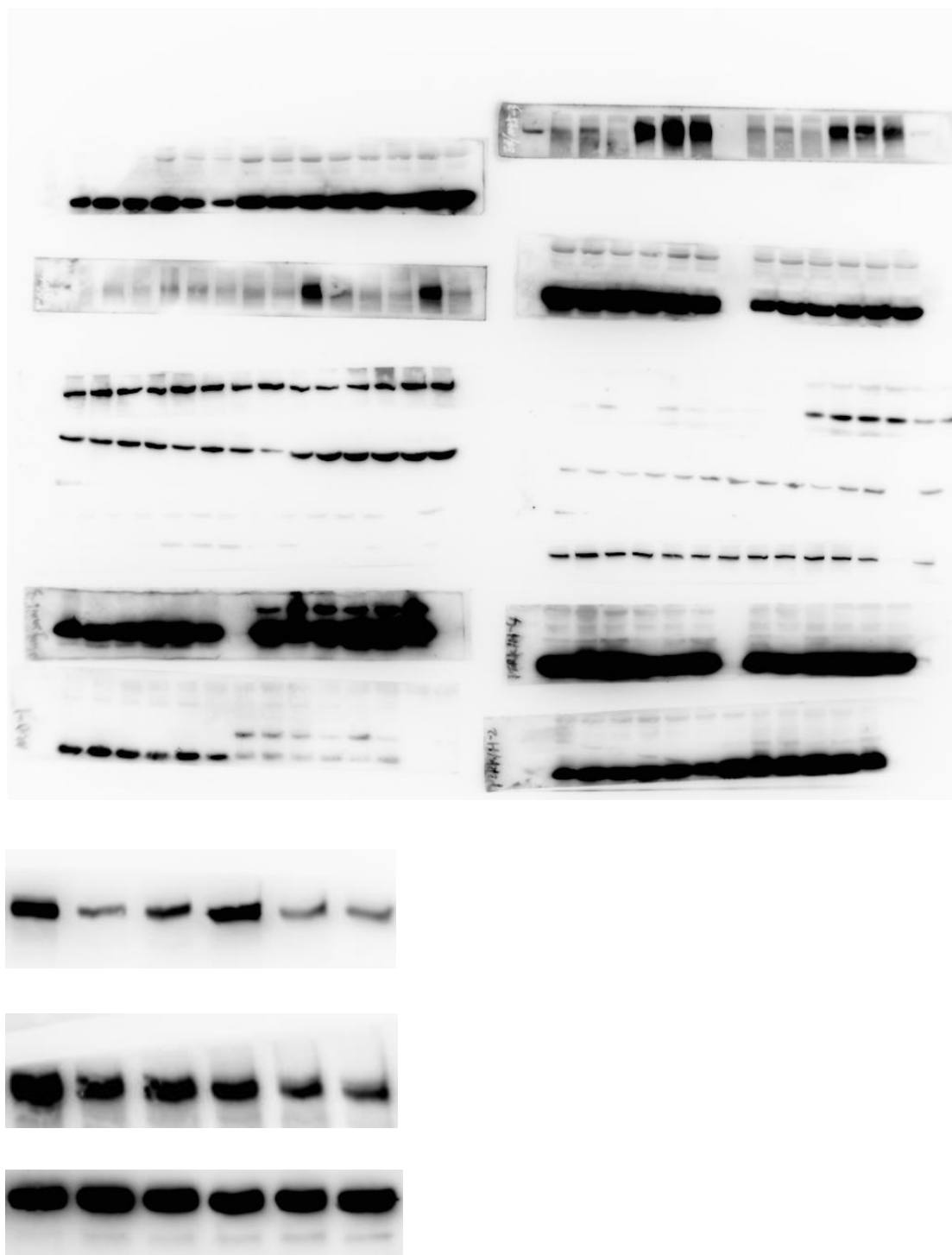

Fig6A

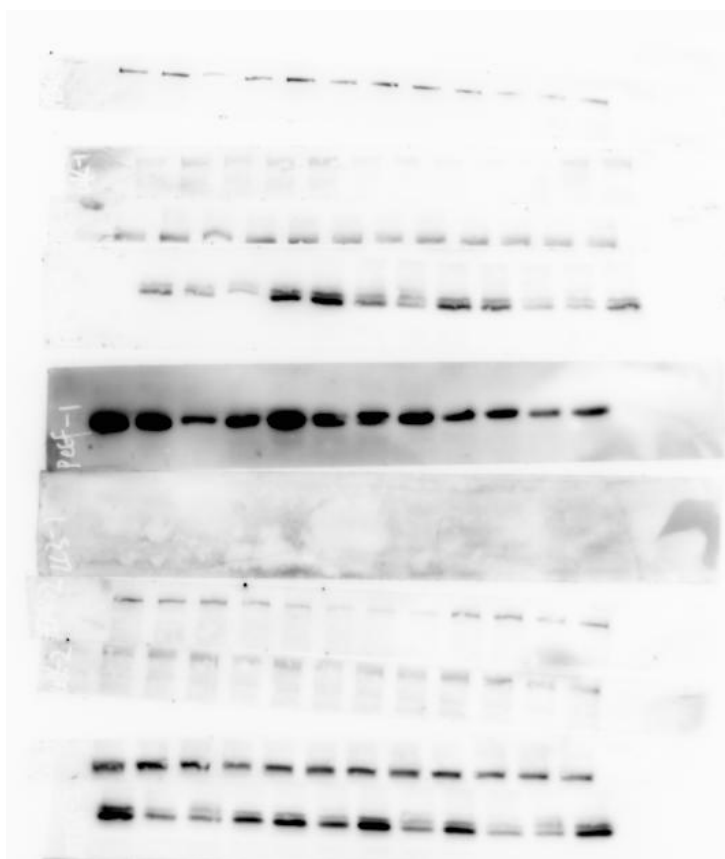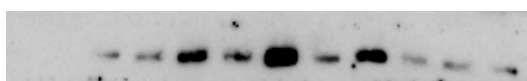

ATG5-1

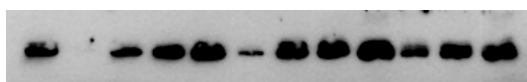

ATG5-2

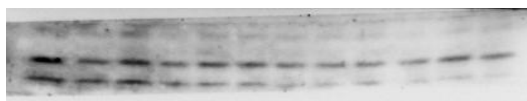

LC3

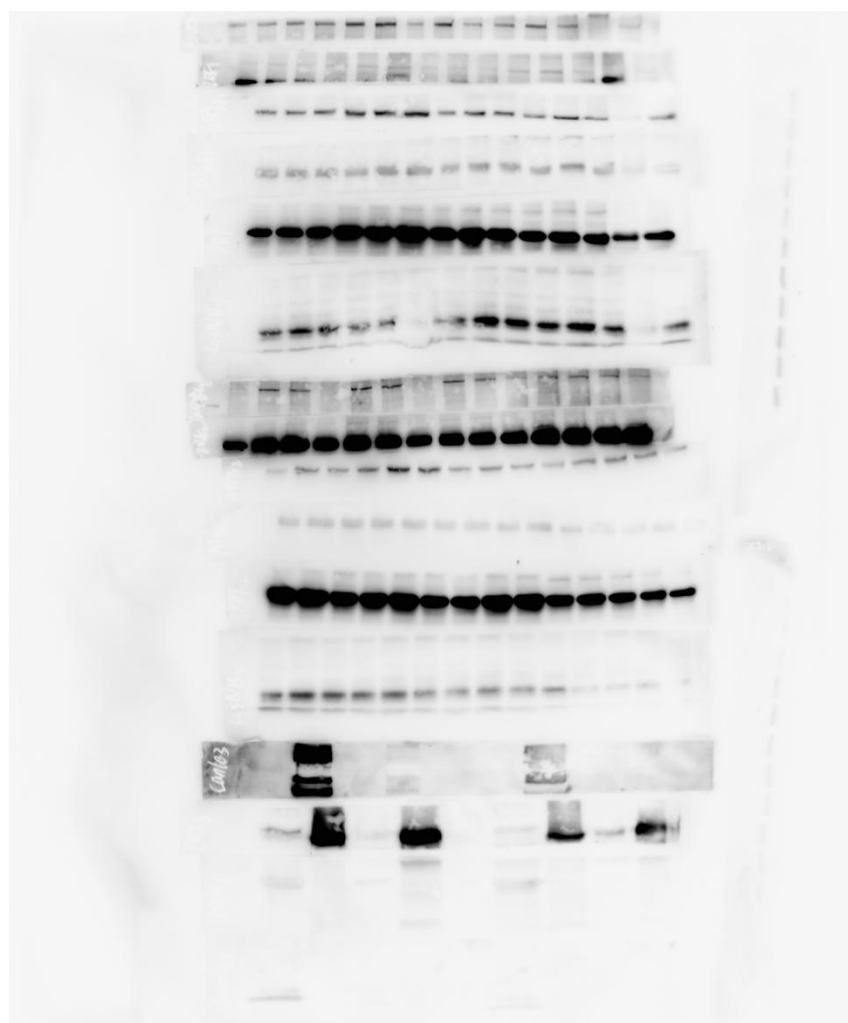

Supplement: Supplementary file 9 — Original Data of WB [file 41419_2026_8914_MOESM9_ESM.pdf]
