# Supplementary material for: GATA6-CRT axis promotes stress-associated autophagy, EMT, and stemness-associated traits in pancreatic cancer
Source: Cell Death Dis. 2026 Jun 4;17(1):610. doi: 10.1038/s41419-026-08914-8 (PMC13323382; doi:10.1038/s41419-026-08914-8)
Supplement: Supplementary file 10 — Original Data of PCR [file 41419_2026_8914_MOESM10_ESM.pdf]

Figure6B

	Cell line	GATA6-CT	GAPDH-CT		CRT-CT	GAPDH-CT	△CT1-△CT2	–（△CT1-△CT2）	Ratio
Ctrl	PANC-1	21.71	17.17	Ctrl	21.71	17.17	0	0	**1.00**
GATA6siRNA1	PANC-1	22.42	16.25	Ctrl	21.71	17.17	1.63	-1.63	**0.32**
GATA6siRNA2	PANC-1	22.31	16.31	Ctrl	21.71	17.17	1.46	-1.46	**0.36**
		CRT-CT	GAPDH-CT		CRT-CT	GAPDH-CT			
Ctrl	PANC-1	18.16	17.17	Ctrl	18.16	17.17	0	0	**1.00**
GATA6siRNA1	PANC-1	18.49	16.25	Ctrl	18.16	17.17	1.25	-1.25	**0.42**
GATA6siRNA2	PANC-1	18.26	16.31	Ctrl	18.16	17.17	0.96	-0.96	**0.51**

Repeat 3 times Capan-2

GATA6 relative mRNA expresiso		
Ctrl	si1-GATA6	si2-GATA6
1	0.321	0.361
1	0.231	0.412
1	0.181	0.301

CRT relative mRNA expresion		
Ctrl	si1-GATA6	si2-GATA6
1	0.421	0.511
1	0.352	0.422
1	0.315	0.389

	Cell line	GATA6-CT	GAPDH-CT		CRT-CT	GAPDH-CT	△CT1-△CT2	–（△CT1-△CT2）	Ratio
Ctrl	Panc02	23.71	18.17	Ctrl	23.71	18.17	0	0	**1.00**
GATA6siRNA1	Panc02	26.05	19.25	Ctrl	23.71	18.17	1.26	-1.26	**0.42**
GATA6siRNA1	Panc02	25.18	18.51	Ctrl	23.71	18.17	1.13	-1.13	**0.46**
		CRT-CT	GAPDH-CT		CRT-CT	GAPDH-CT		0	**1.00**
Ctrl	Panc02	21.16	18.17	Ctrl	21.16	18.17	0	0	**1.00**
GATA6siRNA1	Panc02	23.21	19.25	Ctrl	21.16	18.17	0.97	-0.97	**0.51**
GATA6siRNA1	Panc02	22.19	18.51	Ctrl	21.16	18.17	0.69	-0.69	**0.62**

Repeat 3 times Panco2

GATA6 relative mRNA expresiso		
Ctrl	si1-GATA6	si2-GATA6
1	0.421	0.461
1	0.331	0.412
1	0.281	0.501

CRT relative mRNA expresion		
Ctrl	si1-GATA6	si2-GATA6
1	0.511	0.621
1	0.452	0.541
1	0.415	0.579

Supplemental Figure1C

	Cell line	ZBTB26-CT	GAPDH-CT		ZBTB26-C	GAPDH-CT	△CT1-△CT2	–（△CT1-△CT2）	Ratio
Ctrl	PANC-1	25.71	17.87	Ctrl	25.71	17.87	0	0	**1.00**
ZBTB26siRNA1	PANC-1	27.14	18.05	Ctrl	25.71	17.87	1.25	-1.25	**0.42**
ZBTB26siRNA2	PANC-1	27.11	17.61	Ctrl	25.71	17.87	1.66	-1.66	**0.32**
		CRT-CT	GAPDH-CT		CRT-CT	GAPDH-CT			
Ctrl	PANC-1	19.16	17.87	Ctrl	19.16	17.87	0	0	**1.00**
ZBTB26siRNA1	PANC-1	19.40	18.05	Ctrl	19.16	17.87	0.06	-0.06	**0.96**
ZBTB26siRNA2	PANC-1	18.63	17.61	Ctrl	19.16	17.87	-0.27	0.27	**1.21**

Repeat 3 times Capan-2

ZBTB26 relative mRNA expresiso		
Ctrl	si1-ZBTB26	si2-ZBTB26
1	0.421	0.321
1	0.351	0.452

CRT relative mRNA expresion		
Ctrl	si1-ZBTB26	si2-ZBTB26
1	0.961	1.211
1	1.112	0.822

1	0.281	0.515

1	1.201	0.989
